# Supplementary material for: An axonemal intron splicing program sustains Plasmodium male development
Source: Nat Commun. 2024 Jun 1;15:4697. doi: 10.1038/s41467-024-49002-9 (PMC11144265; doi:10.1038/s41467-024-49002-9)
Supplement: Supplementary file 1 — Supplementary Information [file 41467_2024_49002_MOESM1_ESM.pdf]

Supplemental Information

An axonemal intron splicing program sustains *Plasmodium* male development

Jiepeng Guan[1,#], Peijia Wu[1,#], Xiaoli Mo[1,#], Xiaolong Zhang[3,#], Wenqi Liang[1], Xiaoming Zhang[1], Lubin Jiang[3,*], Jian Li[1,*], Huiting Cui[1,*] and Jing Yuan[1,2,*]

1. Supplementary Figures 1-13 and figure legends

2. Supplementary Table 1. List of genetically modified parasite strains used in this study

3. Supplementary Table 2. Oligonucleotides and primers used in this study

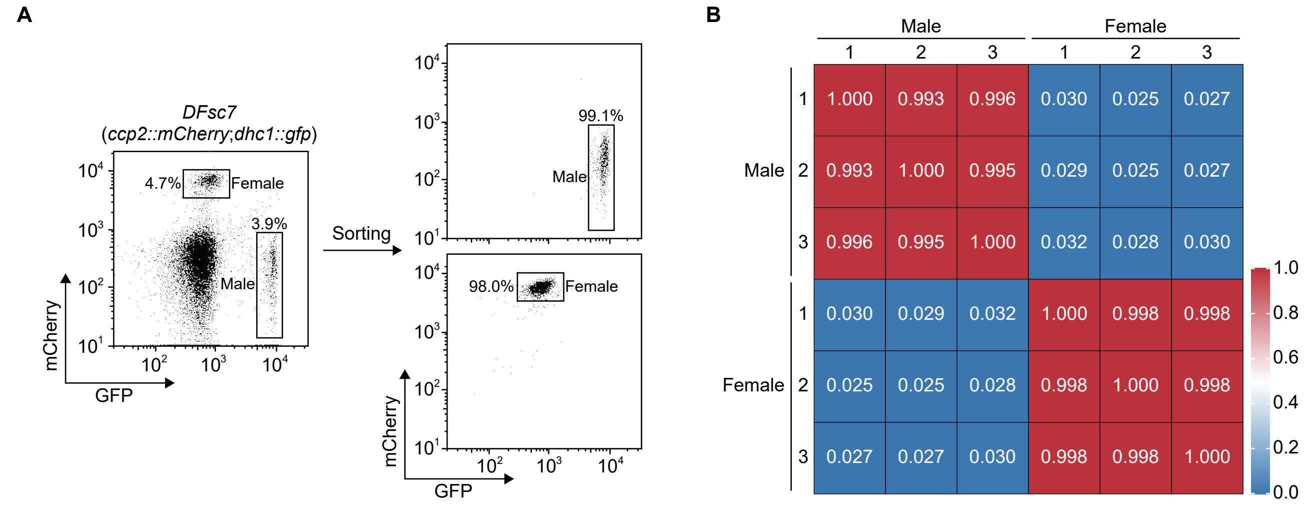
A
DFsc7
(ccp2::mCherry;dhc1::gfp)
4.7%
Female
3.9%
Male
mCherry
GFP
Sorting
99.1%
Male
98.0%
Female
mCherry
GFP
B
Male
Female
1 2 3 1 2 3
Male 1: 1.000 0.993 0.996 0.030 0.025 0.027
Male 2: 0.993 1.000 0.995 0.029 0.025 0.027
Male 3: 0.996 0.995 1.000 0.032 0.028 0.030
Female 1: 0.030 0.029 0.032 1.000 0.998 0.998
Female 2: 0.025 0.025 0.028 0.998 1.000 0.998
Female 3: 0.027 0.027 0.030 0.998 0.998 1.000
1.0
0.8
0.6
0.4
0.2
0.0

Supplementary Figure 1. Purification of male and female gametocytes of the *P. yoelii* parasite for transcriptome analysis

A. Purification of male (GFP+) and female (mCherry+) gametocytes from a *P. yoelii* parasite reporter line *DFsc7* using flow cytometry sorting. The purity of gametocytes was shown. Representative from three independent experiments.

B. Pearson's correlation coefficient analysis of global gene expression between male and female gametocytes based on RNA-seq data with three biological replicates.

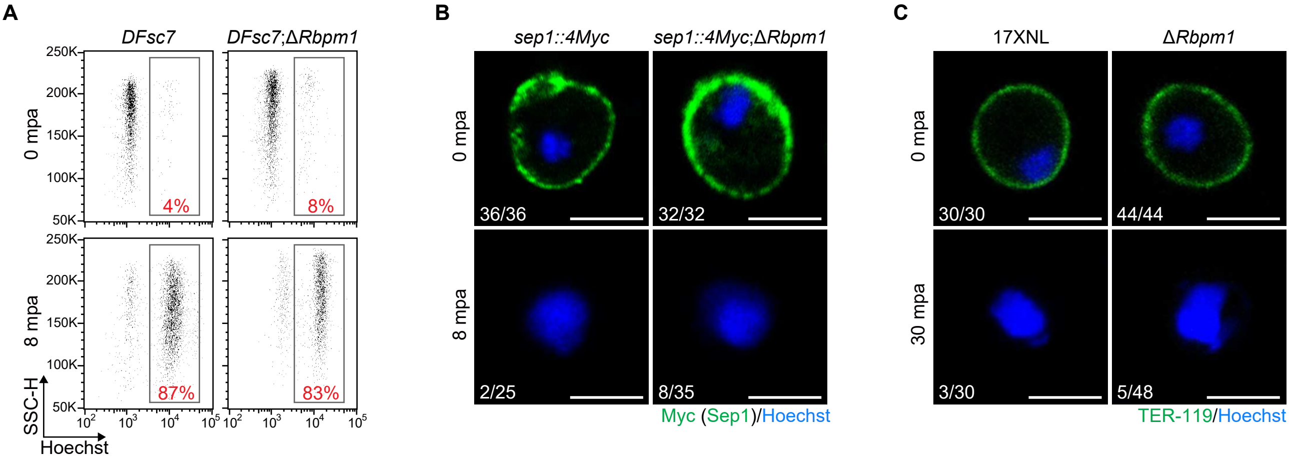
A
DFsc7
DFsc7;ΔRbpm1
0 mpa
8 mpa
4%
8%
87%
83%
250K
200K
150K
100K
50K
SSC-H
Hoechst
10^2
10^3
10^4
10^5
B
sep1::4Myc
sep1::4Myc;ΔRbpm1
36/36
32/32
2/25
8/35
Myc (Sep1)/Hoechst
C
17XNL
ΔRbpm1
30 mpa
30/30
44/44
3/30
5/48
TER-119/Hoechst

Supplementary Figure 2. Normal ability of genome replication and erythrocyte rupture for the RBPm1-null parasite during male gametogenesis

A. Flow cytometry analysis of genomic DNA content in male gametocytes during gametogenesis. The *DFsc7*;Δ*Rbpm1* is a *DFsc7*-derived RBPm1-null parasite line. Male gametocytes (GFP+) were gated, and genomic DNA content was measured based on the Hoechst 33342 fluorescence intensity. Representative for three independent experiments.

B. IFA detection of the parasitophorous vacuole membrane (PVM) rupture. SEP1 protein is a marker for PVM. Male gametocytes from the *sep1::4Myc* parasite and the derived RBPm1-null parasite *sep1::4Myc*;Δ*Rbpm1* were analyzed. Representative for three independent experiments. Scale bars: 5 µm.

C. IFA detection of the erythrocyte plasma membrane (EM) rupture. TER-119 protein is a marker of mouse EM. Male gametocytes from the 17XNL and Δ*Rbpm1* parasites were stained with anti-TER-119 antibody. Representative for three independent experiments. Scale bars: 5 µm.

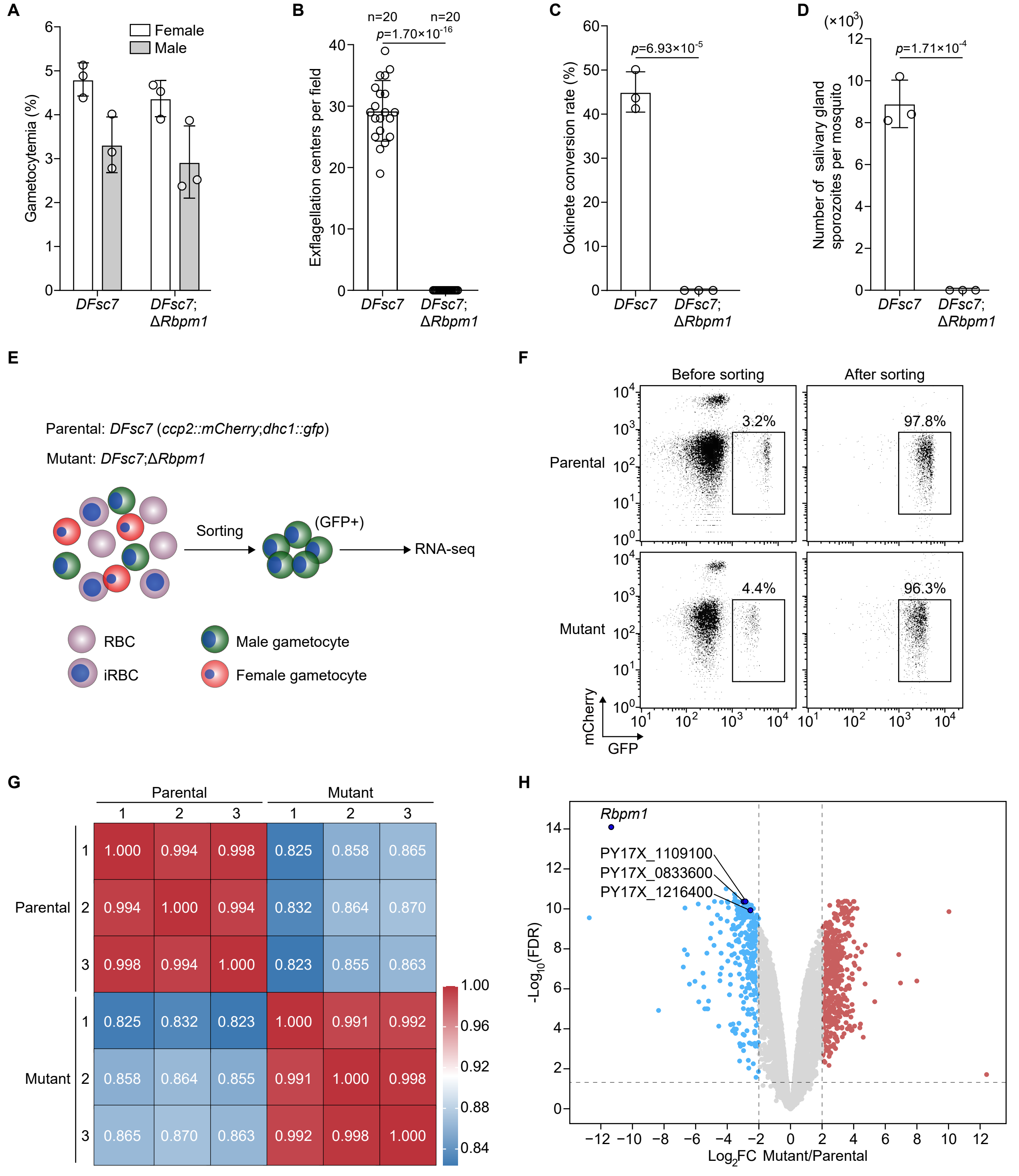
A
Female
Male
Gametocytemia (%)
DFsc7
DFsc7;
ΔRbpm1
B
n=20
n=20
p=1.70×10-16
Exflagellation centers per field
DFsc7
DFsc7;
ΔRbpm1
C
p=6.93×10-5
Ookinete conversion rate (%)
DFsc7
DFsc7;
ΔRbpm1
D
(×103)
p=1.71×10-4
Number of salivary gland sporozoites per mosquito
DFsc7
DFsc7;
ΔRbpm1
E
Parental: DFsc7 (ccp2::mCherry;dhc1::gfp)
Mutant: DFsc7;ΔRbpm1
Sorting
(GFP+)
RNA-seq
RBC
iRBC
Male gametocyte
Female gametocyte
F
Before sorting
After sorting
Parental
Mutant
3.2%
97.8%
4.4%
96.3%
mCherry
GFP
G
Parental
Mutant
1 2 3 1 2 3
Parental 1: 1.000 0.994 0.998 0.825 0.858 0.865
Parental 2: 0.994 1.000 0.994 0.832 0.864 0.870
Parental 3: 0.998 0.994 1.000 0.823 0.855 0.863
Mutant 1: 0.825 0.832 0.823 1.000 0.991 0.992
Mutant 2: 0.858 0.864 0.855 0.991 1.000 0.998
Mutant 3: 0.865 0.870 0.863 0.992 0.998 1.000
1.00
0.96
0.92
0.88
0.84
H
Rbpm1
PY17X_1109100
PY17X_0833600
PY17X_1216400
-Log10(FDR)
Log2FC Mutant/Parental

Supplementary Figure 3. Generation, purification and transcriptome analysis of male gametocytes with RBPm1 deficiency

A-D. Phenotype analysis of *DFsc7* (parental) and *DFsc7*;Δ*Rbpm1* (mutant) parasite lines, including gametocyte formation (**A**), male gamete formation (**B**), ookinete formation *in vitro* (**C**), and salivary gland sporozoite in mosquitoes (**D**). Data are means ± SEM of three independent experiments, two-sided *t*-test.

E. Flowchart showing the purification of male gametocytes (green, GFP+) from both *DFsc7* and *DFsc7*;Δ*Rbpm1* parasites for transcriptome analysis via RNA-seq.

F. Flow cytometry detection of male gametocytes (GFP+) before and after sorting, with indicated purity. Representative results from three independent experiments.

G. A heatmap showing the Pearson correlation coefficient between *DFsc7* (parental) and *DFsc7*;Δ*Rbpm1* (mutant) male gametocyte RNA-seq data.

H. A volcano plot showing the differentially expressed genes in male gametocytes between the *DFsc7* (parental) and *DFsc7*;Δ*Rbpm1* (mutant) lines. The threshold for the $\log_2$ fold change ($\log_2$FC) and false discovery rate (FDR) are ±2 and 0.05, respectively. There are 481 genes up-regulated and 295 genes down-regulated in male gametocytes after loss of RBPm1. Three down-regulated genes (PY17X_1109100, PY17X_0833600, and PY17X_1216400), which exhibited intron retention in further study, are highlighted.

A

26 genes with intron retention

Molecular function	Gene ID	Total number of introns in gene	# of the retained intron	Length of the retained intron (bp)	Male transcription (normalized counts)	Female transcription (normalized counts)	Ratio (Male/Female)
Basal body & axoneme assembly	*kinesin8b* (PY17X_0204100)	4	1	239	5148	2	3217
	PF16 (PY17X_0919000)	1	1	276	580	38	15
Axoneme motility	*dhc6* (PY17X_0603800)	33	20	241	842	5	160
	dhc7 (PY17X_0510800)	10	7	235	1070	3	318
	dlc1 (PY17X_1241500)	5	4	193	296	68	4
	dlc2 (PY17X_0302800)	3	1	195	503	19	27
	drc1 (PY17X_0721100)	9	2, 3	179, 150	597	93	6
	dbc (PY17X_1333900)	5	1	772	2059	40	51
Function unknown	*md2* (PY17X_1450400)	1	1	505	1534	53	29
	PY17X_1109100	3	1	353	1662	55	30
	PY17X_0521800	16	1	278	777	38	20
	PY17X_1311800	13	5	248	1342	3	446
	PY17X_1323900	1	1	322	710	103	7
	PY17X_1357300	7	5, 7	212, 143	2644	4	630
	PY17X_1335600	1	1	338	1320	3	417
	PY17X_1452900	13	1	367	1686	6	295
	PY17X_1122300	2	1	240	161	10	15
	PY17X_0523500	5	2, 3	192, 318	348	1	533
	PY17X_0508900	7	5	380	485	10	49
	PY17X_1320300	3	2	183	195	24	8
	PY17X_0833600	2	2	302	526	12	46
	PY17X_1341200	5	1	208	731	1	803
	PY17X_1305400	15	11, 12	261, 330	1583	14	116
	PY17X_0415900	13	13	171	431	2	201
	PY17X_0105800	1	1	193	107	3	33
	PY17X_1216400	10	5	134	712	1	596

B

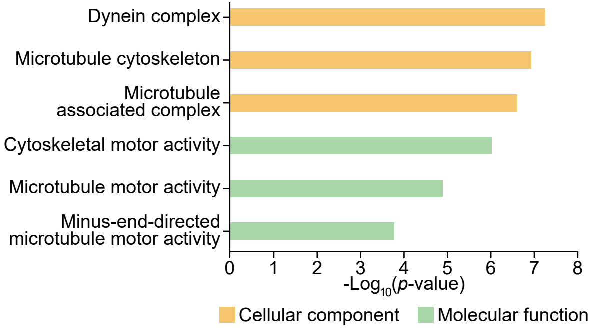

Supplementary Figure 4. Expression and function information of 26 intron-retained genes identified in the RBPm1-null male gametocytes

A. List of 26 genes with intron retention detected in the RBPm1-null male gametocytes. Among them, 22 genes had one retained intron while 4 genes (*drc1*, PY17X_1357300, PY17X_0523500, and PY17X_1305400) possessed two retained introns after loss of RBPm1. Information including the protein function, gene ID, exon-intron structure, retained intron, and gender transcription, is provided. Gender transcription (TMM normalized counts) were from the gametocyte transcriptome data in this study.

B. Gene ontology enrichment analysis of the 26 genes indicates male-specific or preferential biological processes. Hypergeometric test was applied.

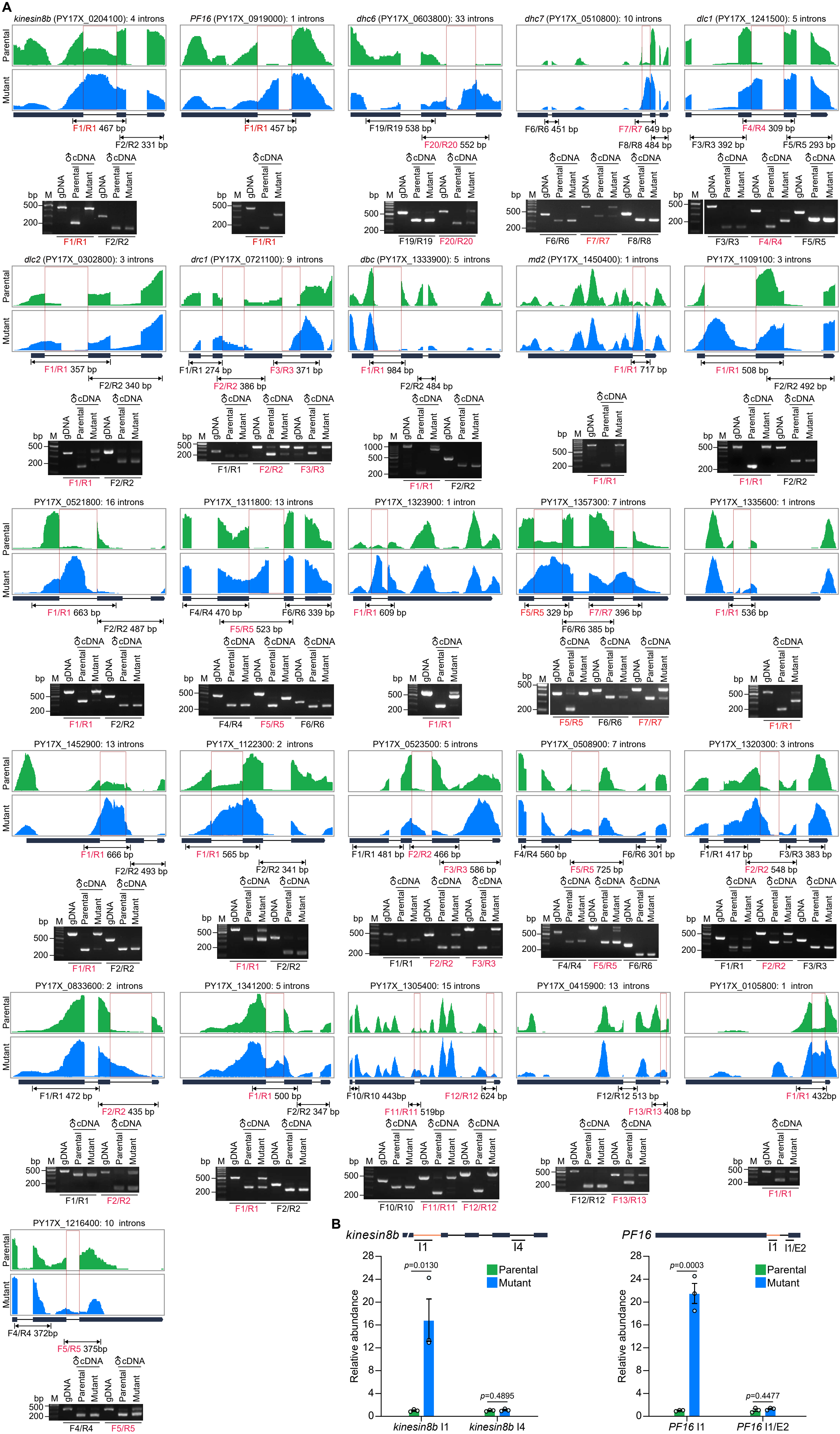
A
kinesin8b (PY17X_0204100): 4 introns
PF16 (PY17X_0919000): 1 introns
dhc6 (PY17X_0603800): 33 introns
dhc7 (PY17X_0510800): 10 introns
dlc1 (PY17X_1241500): 5 introns
Parental
Mutant
F1/R1 467 bp
F2/R2 331 bp
F1/R1 457 bp
F19/R19 538 bp
F20/R20 552 bp
F6/R6 451 bp
F7/R7 649 bp
F8/R8 484 bp
F4/R4 309 bp
F3/R3 392 bp
F5/R5 293 bp
♂cDNA
bp
M
gDNA
Parental
Mutant
500
200
dlc2 (PY17X_0302800): 3 introns
drc1 (PY17X_0721100): 9 introns
dbc (PY17X_1333900): 5 introns
md2 (PY17X_1450400): 1 introns
PY17X_1109100: 3 introns
F1/R1 357 bp
F2/R2 340 bp
F1/R1 274 bp
F2/R2 386 bp
F3/R3 371 bp
F1/R1 984 bp
F2/R2 484 bp
F1/R1 717 bp
F1/R1 508 bp
F2/R2 492 bp
1000
PY17X_0521800: 16 introns
PY17X_1311800: 13 introns
PY17X_1323900: 1 intron
PY17X_1357300: 7 introns
PY17X_1335600: 1 introns
F1/R1 663 bp
F2/R2 487 bp
F4/R4 470 bp
F6/R6 339 bp
F5/R5 523 bp
F1/R1 609 bp
F5/R5 329 bp
F7/R7 396 bp
F6/R6 385 bp
F1/R1 536 bp
PY17X_1452900: 13 introns
PY17X_1122300: 2 introns
PY17X_0523500: 5 introns
PY17X_0508900: 7 introns
PY17X_1320300: 3 introns
F1/R1 666 bp
F2/R2 493 bp
F1/R1 565 bp
F2/R2 341 bp
F1/R1 481 bp
F2/R2 466 bp
F3/R3 586 bp
F4/R4 560 bp
F6/R6 301 bp
F5/R5 725 bp
F1/R1 417 bp
F3/R3 383 bp
F2/R2 548 bp
PY17X_0833600: 2 introns
PY17X_1341200: 5 introns
PY17X_1305400: 15 introns
PY17X_0415900: 13 introns
PY17X_0105800: 1 intron
F1/R1 472 bp
F2/R2 435 bp
F1/R1 500 bp
F2/R2 347 bp
F10/R10 443bp
F11/R11 519bp
F12/R12 624 bp
F12/R12 513 bp
F13/R13 408 bp
F1/R1 432bp
PY17X_1216400: 10 introns
F4/R4 372bp
F5/R5 375bp
B
kinesin8b
I1
I4
PF16
I1/E2
Relative abundance
Parental
Mutant
p=0.0130
p=0.4895
p=0.0003
p=0.4477
0
4
8
12
16
20
24
28
kinesin8b I1
kinesin8b I4
PF16 I1
PF16 I1/E2

Supplementary Figure 5. Verification of intron retention for 30 introns at 26 genes in the RBPm1-null male gametocytes

A. RT-PCR confirmation of intron retention in the 26 genes after loss of RBPm1. For each gene, the mapped views of the RNA-seq results (*DFsc7* in green and *DFsc7*;Δ*Rbpm1* in blue, representative for three biological replicates) and the exon-intron structure (black) are shown in the upper panels. Intron retention is highlighted with red boxes. The primers (F or R) designed for detecting the intron via RT-PCR and the expected PCR products are shown. RT-PCR analysis using the genomic DNA (gDNA) from 17XNL parasite, complementary DNA (cDNA) from male gametocytes of parental and mutant parasites showed the intron retention (red) and intron splicing (black).

B. RT-qPCR confirmation of intron retention in the *kinesin8b* and *PF16* genes after loss of RBPm1. The top schematic of gene exon-intron structure shows the positions of the retained intron (orange line) and the RT-qPCR amplicon. RT-qPCR analysis using cDNA from male gametocytes of parental and mutant parasites showed the retention of *kinesin8b* intron1 and *PF16* intron1. Data are means ± SEM from three independent experiments, two-sided *t*-test.

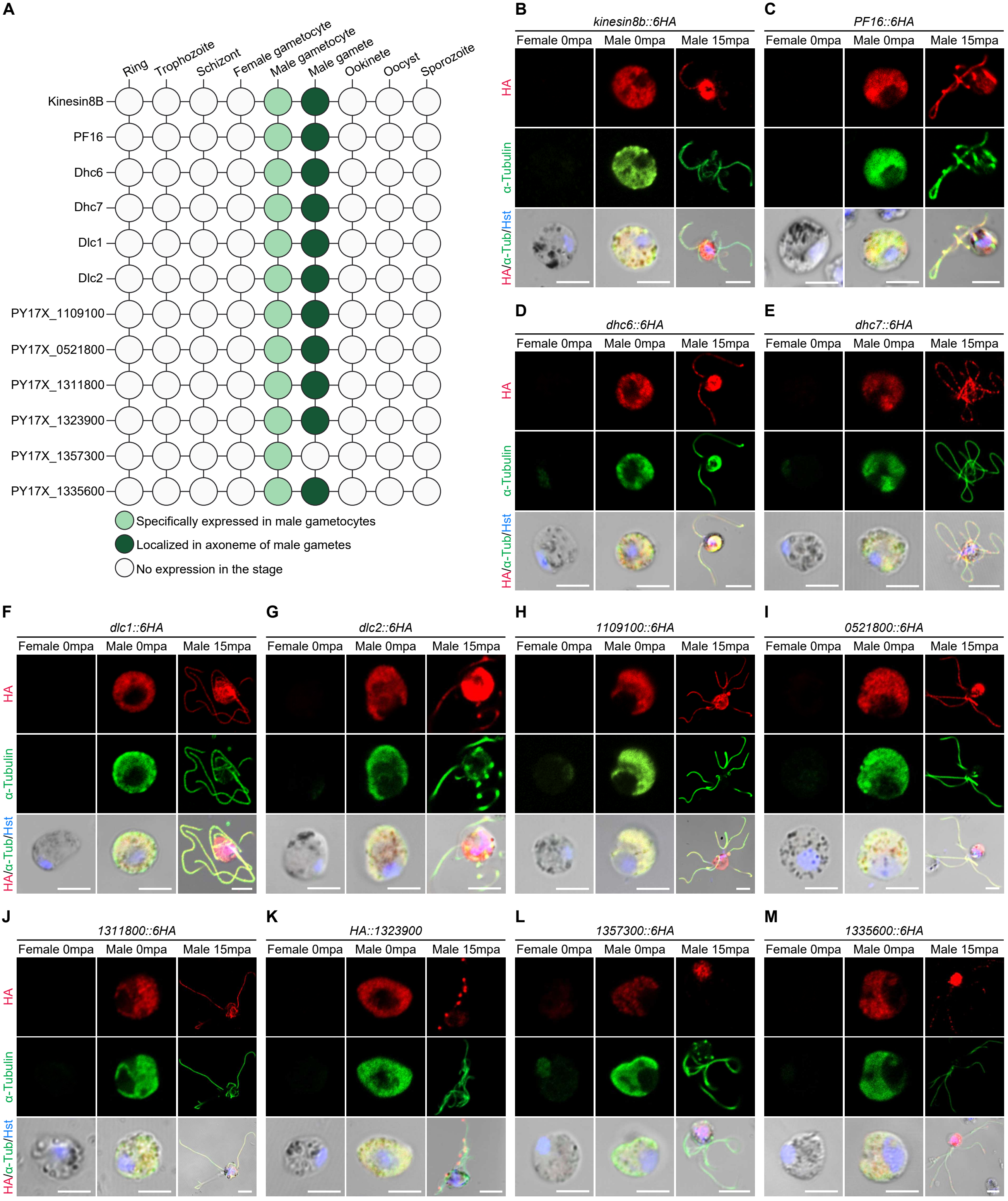
A
Ring
Trophozoite
Schizont
Female gametocyte
Male gametocyte
Male gamete
Ookinete
Oocyst
Sporozoite
Kinesin8B
PF16
Dhc6
Dhc7
Dlc1
Dlc2
PY17X_1109100
PY17X_0521800
PY17X_1311800
PY17X_1323900
PY17X_1357300
PY17X_1335600
Specifically expressed in male gametocytes
Localized in axoneme of male gametes
No expression in the stage
B
kinesin8b::6HA
C
PF16::6HA
D
dhc6::6HA
E
dhc7::6HA
F
dlc1::6HA
G
dlc2::6HA
H
1109100::6HA
I
0521800::6HA
J
1311800::6HA
K
HA::1323900
L
1357300::6HA
M
1335600::6HA
Female 0mpa
Male 0mpa
Male 15mpa
HA
α-Tubulin
HA/α-Tub/Hst

Supplementary Figure 6. RBPm1-regulating genes encode axoneme-associated proteins

A. Summary of protein stage expression and localization of 12 selected intron-retained genes in the *P. yoelii*. These genes include 6 annotated genes (*kinesin8b*, *PF16*, *dhc6*, *dhc7*, *dlc1*, *dlc2*) and 6 unannotated genes (PY17X_1109100, PY17X_0521800, PY17X_1311800, PY17X_1323900, PY17X_1357300, PY17X_1335600). Each gene was endogenously tagged at the N- or C-terminus with a 6HA in the 17XNL, generating the HA-tagged lines for protein expression and localization analysis. Due to the space limit, only the results of protein expression at the gametocytes were shown in **B-M.**

B-M. Protein expression and localization analysis of the 12 intron-retaining genes in gametocytes. IFA of the HA-tagged target protein and α-Tubulin in female gametocytes (0 mpa) and male gametocytes (0 and 15 mpa). Representative results from two independent experiments. Scale bars: 5 µm.

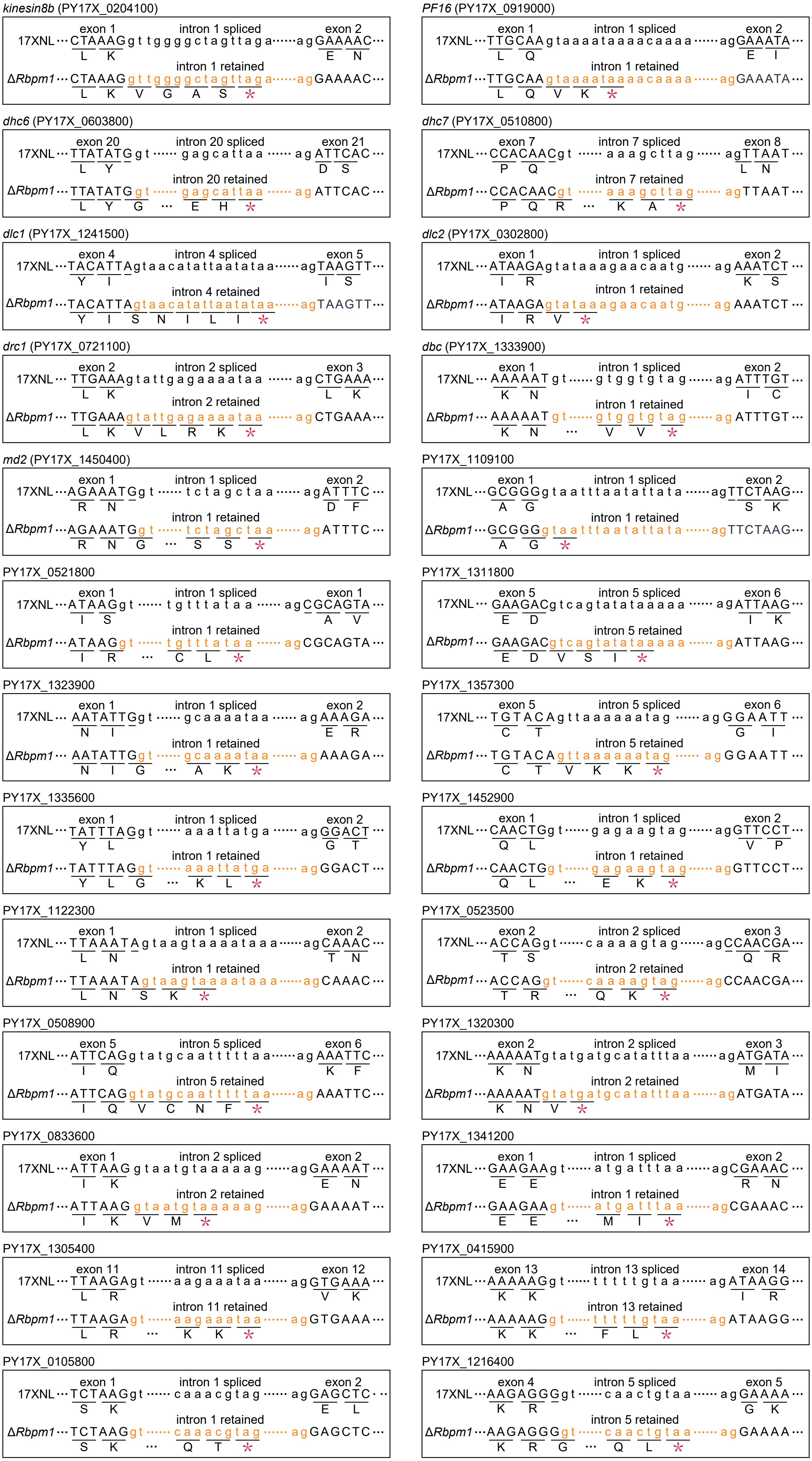

kinesin8b (PY17X_0204100)
exon 1 intron 1 spliced exon 2
17XNL ··· CTAAAGgttggggctagttaga······agGAAAAC···
L K E N
intron 1 retained
ΔRbpm1 ··· CTAAAGgttggggctagttaga······agGAAAAC···
L K V G A S *
PF16 (PY17X_0919000)
exon 1 intron 1 spliced exon 2
17XNL ··· TTGCAAgtaaaataaaacaaaa······agGAAATA···
L Q E I
intron 1 retained
ΔRbpm1 ··· TTGCAAgtaaaataaaacaaaa······agGAAATA···
L Q V K *
dhc6 (PY17X_0603800)
exon 20 intron 20 spliced exon 21
17XNL ··· TTATATGgt······gagcattaa······agATTCAC···
L Y D S
intron 20 retained
ΔRbpm1 ··· TTATATGgt······gagcattaa······agATTCAC···
L Y G ··· E H *
dhc7 (PY17X_0510800)
exon 7 intron 7 spliced exon 8
17XNL ··· CCACAACgt······aaagcttag······agTTAAT···
P Q L N
intron 7 retained
ΔRbpm1 ··· CCACAACgt······aaagcttag······agTTAAT···
P Q R ··· K A *
dlc1 (PY17X_1241500)
exon 4 intron 4 spliced exon 5
17XNL ··· TACATTAgtaacatattaatataa······agTAAGTT···
Y I I S
intron 4 retained
ΔRbpm1 ··· TACATTAgtaacatattaatataa······agTAAGTT···
Y I S N I L I *
dlc2 (PY17X_0302800)
exon 1 intron 1 spliced exon 2
17XNL ··· ATAAGAgtataaagaacaatg······agAAATCT···
I R K S
intron 1 retained
ΔRbpm1 ··· ATAAGAgtataaagaacaatg······agAAATCT···
I R V *
drc1 (PY17X_0721100)
exon 2 intron 2 spliced exon 3
17XNL ··· TTGAAAgtattgagaaaataa······agCTGAAA···
L K L K
intron 2 retained
ΔRbpm1 ··· TTGAAAgtattgagaaaataa······agCTGAAA···
L K V L R K *
dbc (PY17X_1333900)
exon 1 intron 1 spliced exon 2
17XNL ··· AAAAATgt······gtggtgtag······agATTTGT···
K N I C
intron 1 retained
ΔRbpm1 ··· AAAAATgt······gtggtgtag······agATTTGT···
K N ··· V V *
md2 (PY17X_1450400)
exon 1 intron 1 spliced exon 2
17XNL ··· AGAAATGgt······tctagctaa······agATTTC···
R N D F
intron 1 retained
ΔRbpm1 ··· AGAAATGgt······tctagctaa······agATTTC···
R N G ··· S S *
PY17X_1109100
exon 1 intron 1 spliced exon 2
17XNL ··· GCGGGgtaatttaatattata······agTTCTAAG···
A G S K
intron 1 retained
ΔRbpm1 ··· GCGGGgtaatttaatattata······agTTCTAAG···
A G *
PY17X_0521800
exon 1 intron 1 spliced exon 1
17XNL ··· ATAAGgt······tgtttataa······agCGCAGTA···
I S A V
intron 1 retained
ΔRbpm1 ··· ATAAGgt······tgtttataa······agCGCAGTA···
I R ··· C L *
PY17X_1311800
exon 5 intron 5 spliced exon 6
17XNL ··· GAAGACgtcagtatataaaaa······agATTAAG···
E D I K
intron 5 retained
ΔRbpm1 ··· GAAGACgtcagtatataaaaa······agATTAAG···
E D V S I *
PY17X_1323900
exon 1 intron 1 spliced exon 2
17XNL ··· AATATTGgt······gcaaaataa······agAAAGA···
N I E R
intron 1 retained
ΔRbpm1 ··· AATATTGgt······gcaaaataa······agAAAGA···
N I G ··· A K *
PY17X_1357300
exon 5 intron 5 spliced exon 6
17XNL ··· TGTACAgttaaaaaatag······agGGAATT···
C T G I
intron 5 retained
ΔRbpm1 ··· TGTACAgttaaaaaatag······agGGAATT···
C T V K K *
PY17X_1335600
exon 1 intron 1 spliced exon 2
17XNL ··· TATTTAGgt······aaattatga······agGGACT···
Y L G T
intron 1 retained
ΔRbpm1 ··· TATTTAGgt······aaattatga······agGGACT···
Y L G ··· K L *
PY17X_1452900
exon 1 intron 1 spliced exon 2
17XNL ··· CAACTGgt······gagaagtag······agGTTCCT···
Q L V P
intron 1 retained
ΔRbpm1 ··· CAACTGgt······gagaagtag······agGTTCCT···
Q L ··· E K *
PY17X_1122300
exon 1 intron 1 spliced exon 2
17XNL ··· TTAAATAgtaagtaaaataaa······agCAAAC···
L N T N
intron 1 retained
ΔRbpm1 ··· TTAAATAgtaagtaaaataaa······agCAAAC···
L N S K *
PY17X_0523500
exon 2 intron 2 spliced exon 3
17XNL ··· ACCAGgt······caaaagtag······agCCAACGA···
T S Q R
intron 2 retained
ΔRbpm1 ··· ACCAGgt······caaaagtag······agCCAACGA···
T R ··· Q K *
PY17X_0508900
exon 5 intron 5 spliced exon 6
17XNL ··· ATTCAGgtatgcaattttttaa······agAAATTC···
I Q K F
intron 5 retained
ΔRbpm1 ··· ATTCAGgtatgcaattttttaa······agAAATTC···
I Q V C N F *
PY17X_1320300
exon 2 intron 2 spliced exon 3
17XNL ··· AAAAATgtatgatgcatatttaa······agATGATA···
K N M I
intron 2 retained
ΔRbpm1 ··· AAAAATgtatgatgcatatttaa······agATGATA···
K N V *
PY17X_0833600
exon 1 intron 2 spliced exon 2
17XNL ··· ATTAAGgtaatgtaaaaag······agGAAAAT···
I K E N
intron 2 retained
ΔRbpm1 ··· ATTAAGgtaatgtaaaaag······agGAAAAT···
I K V M *
PY17X_1341200
exon 1 intron 1 spliced exon 2
17XNL ··· GAAGAAgt······atgatttaa······agCGAAAC···
E E R N
intron 1 retained
ΔRbpm1 ··· GAAGAAgt······atgatttaa······agCGAAAC···
E E ··· M I *
PY17X_1305400
exon 11 intron 11 spliced exon 12
17XNL ··· TTAAGAgt······aagaaataa······agGTGAAA···
L R V K
intron 11 retained
ΔRbpm1 ··· TTAAGAgt······aagaaataa······agGTGAAA···
L R ··· K K *
PY17X_0415900
exon 13 intron 13 spliced exon 14
17XNL ··· AAAAAGgt······ttttgtaa······agATAAGG···
K K I R
intron 13 retained
ΔRbpm1 ··· AAAAAGgt······ttttgtaa······agATAAGG···
K K ··· F L *
PY17X_0105800
exon 1 intron 1 spliced exon 2
17XNL ··· TCTAAGgt······caaacgtag······agGAGCTC···
S K E L
intron 1 retained
ΔRbpm1 ··· TCTAAGgt······caaacgtag······agGAGCTC···
S K ··· Q T *
PY17X_1216400
exon 4 intron 5 spliced exon 5
17XNL ··· AAGAGGGgt······caactgtaa······agGAAAA···
K R G K
intron 5 retained
ΔRbpm1 ··· AAGAGGGgt······caactgtaa······agGAAAA···
K R G ··· Q L *

Supplementary Figure 7. Intron retention causes premature stop codons in RBPm1 target transcripts

Amino acid and nucleotide sequence analysis of the retained introns in the 26 genes. For each gene, intron retention (highlighted in orange lowercase letter) creates at least one premature stop codon (red asterisk) at the transcripts in the RBPm1-null parasites.

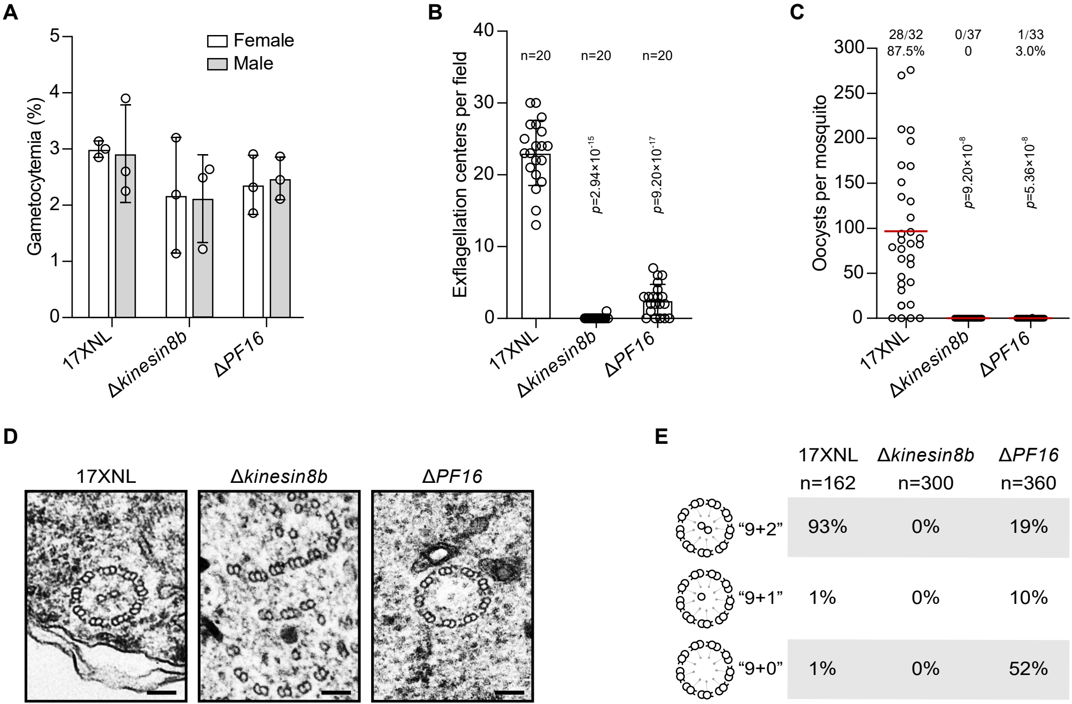
A
Female
Male
Gametocytemia (%)
17XNL
Δkinesin8b
ΔPF16
B
Exflagellation centers per field
n=20
n=20
n=20
p=2.94×10^-15
p=9.20×10^-17
17XNL
Δkinesin8b
ΔPF16
C
Oocysts per mosquito
28/32
87.5%
0/37
0
1/33
3.0%
p=9.20×10^-8
p=5.36×10^-8
17XNL
Δkinesin8b
ΔPF16
D
17XNL
Δkinesin8b
ΔPF16
E
17XNL
n=162
Δkinesin8b
n=300
ΔPF16
n=360
"9+2"
93%
0%
19%
"9+1"
1%
0%
10%
"9+0"
1%
0%
52%

Supplementary Figure 8. Depletion of *kinesin8b* or *PF16* phenocopies RBPm1 deficiency

A. Female and male gametocyte formation in mice. Data are means ± SEM from three independent experiments.

B. Exflagellation center (EC) formation of male gametocytes at 10 mpa. Data are means ± SEM from three independent experiments, two-sided *t*-test.

C. Midgut oocyst formation in mosquitoes at 7 days after blood feeding. x/y at the top represents the number of mosquitoes containing oocysts/the number of dissected mosquitoes, and the percentage represents the infection prevalence of mosquitoes. Red lines show the mean value of oocyst numbers, two-sided Mann–Whitney *U* test. Representative results from two independent experiments.

D. Transmission electron microscopy of axoneme architecture in male gametocytes at 8 mpa. Scale bars: 100 nm.

E. Quantification of axoneme formation in the mutant parasites in **D**. n is the total number of the intact and defective axoneme structures observed in each group. Representative for three independent experiments.

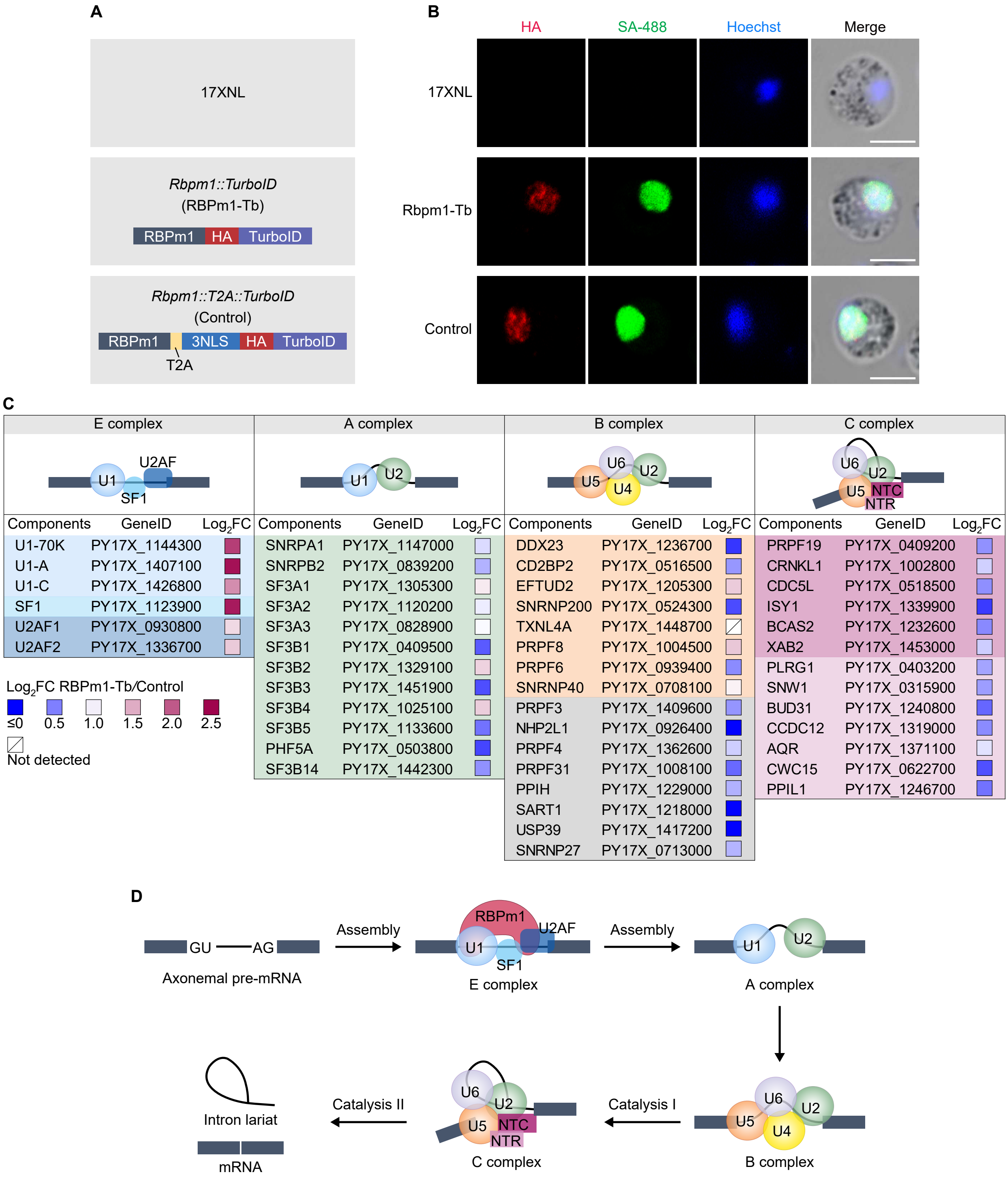
A
17XNL
Rbpm1::TurboID
(RBPm1-Tb)
RBPm1 HA TurboID
Rbpm1::T2A::TurboID
(Control)
RBPm1 3NLS HA TurboID
T2A
B
HA
SA-488
Hoechst
Merge
17XNL
Rbpm1-Tb
Control
C
E complex
U2AF
U1
SF1
Components GeneID Log_2FC
U1-70K PY17X_1144300
U1-A PY17X_1407100
U1-C PY17X_1426800
SF1 PY17X_1123900
U2AF1 PY17X_0930800
U2AF2 PY17X_1336700
Log_2FC RBPm1-Tb/Control
≤0 0.5 1.0 1.5 2.0 2.5
Not detected
A complex
U1
U2
Components GeneID Log_2FC
SNRPA1 PY17X_1147000
SNRPB2 PY17X_0839200
SF3A1 PY17X_1305300
SF3A2 PY17X_1120200
SF3A3 PY17X_0828900
SF3B1 PY17X_0409500
SF3B2 PY17X_1329100
SF3B3 PY17X_1451900
SF3B4 PY17X_1025100
SF3B5 PY17X_1133600
PHF5A PY17X_0503800
SF3B14 PY17X_1442300
B complex
U6
U2
U5
U4
Components GeneID Log_2FC
DDX23 PY17X_1236700
CD2BP2 PY17X_0516500
EFTUD2 PY17X_1205300
SNRNP200 PY17X_0524300
TXNL4A PY17X_1448700
PRPF8 PY17X_1004500
PRPF6 PY17X_0939400
SNRNP40 PY17X_0708100
PRPF3 PY17X_1409600
NHP2L1 PY17X_0926400
PRPF4 PY17X_1362600
PRPF31 PY17X_1008100
PPIH PY17X_1229000
SART1 PY17X_1218000
USP39 PY17X_1417200
SNRNP27 PY17X_0713000
C complex
U6
U2
U5
NTC
NTR
Components GeneID Log_2FC
PRPF19 PY17X_0409200
CRNKL1 PY17X_1002800
CDC5L PY17X_0518500
ISY1 PY17X_1339900
BCAS2 PY17X_1232600
XAB2 PY17X_1453000
PLRG1 PY17X_0403200
SNW1 PY17X_0315900
BUD31 PY17X_1240800
CCDC12 PY17X_1319000
AQR PY17X_1371100
CWC15 PY17X_0622700
PPIL1 PY17X_1246700
D
GU AG
Axonemal pre-mRNA
Assembly
RBPm1
U2AF
U1
SF1
E complex
Assembly
U1
U2
A complex
U6
U2
U5
U4
B complex
Catalysis I
U6
U2
U5
NTC
NTR
C complex
Catalysis II
Intron lariat
mRNA

Supplementary Figure 9. RBPm1 interacts with spliceosome E complex

A. A schematic of two modified parasite lines generated for detecting the RBPm1-interacting proteins in gametocytes by TurboID ligase-based proximity labeling. Endogenous RBPm1 was C-terminally tagged with an HA::TurboID motif by CRISPR-Cas9 in 17XNL, generating the *Rbpm1::TurboID* line. A control line *Rbpm1::T2A::TurboID* was generated, in which a "ribosome skip" T2A was inserted between RBPm1 and 3NLS::HA::TurboID for separated nuclear expression of RBPm1 and TurboID.

B. Co-staining of HA-tagged TurboID ligase (red) and biotinylated proteins (SA-488, green) in male gametocytes of the *Rbpm1::TurboID* and *Rbpm1::T2A::TurboID* lines. Gametocytes incubated with 50 μM biotin at 37°C for 20 minutes were co-stained with the fluorescent-conjugated streptavidin (SA-488) and anti-HA antibody. Scale bars: 5 μm. Representative for three independent experiments.

C. Protein interaction analysis between RBPm1 and spliceosome at different stages of assembly based on the data from TurboID-based proximity labeling and mass spectrometry. The upper panels show the spliceosome complexes from early to later stages during assembly, including E, A, B, and C complexes. The lower panels show the enrichment level (log_2FC value) of protein components in different spliceosome complexes.

D. Proposed model showing RBPm1 interaction with the early spliceosome E complex for intron splicing of axonemal genes.

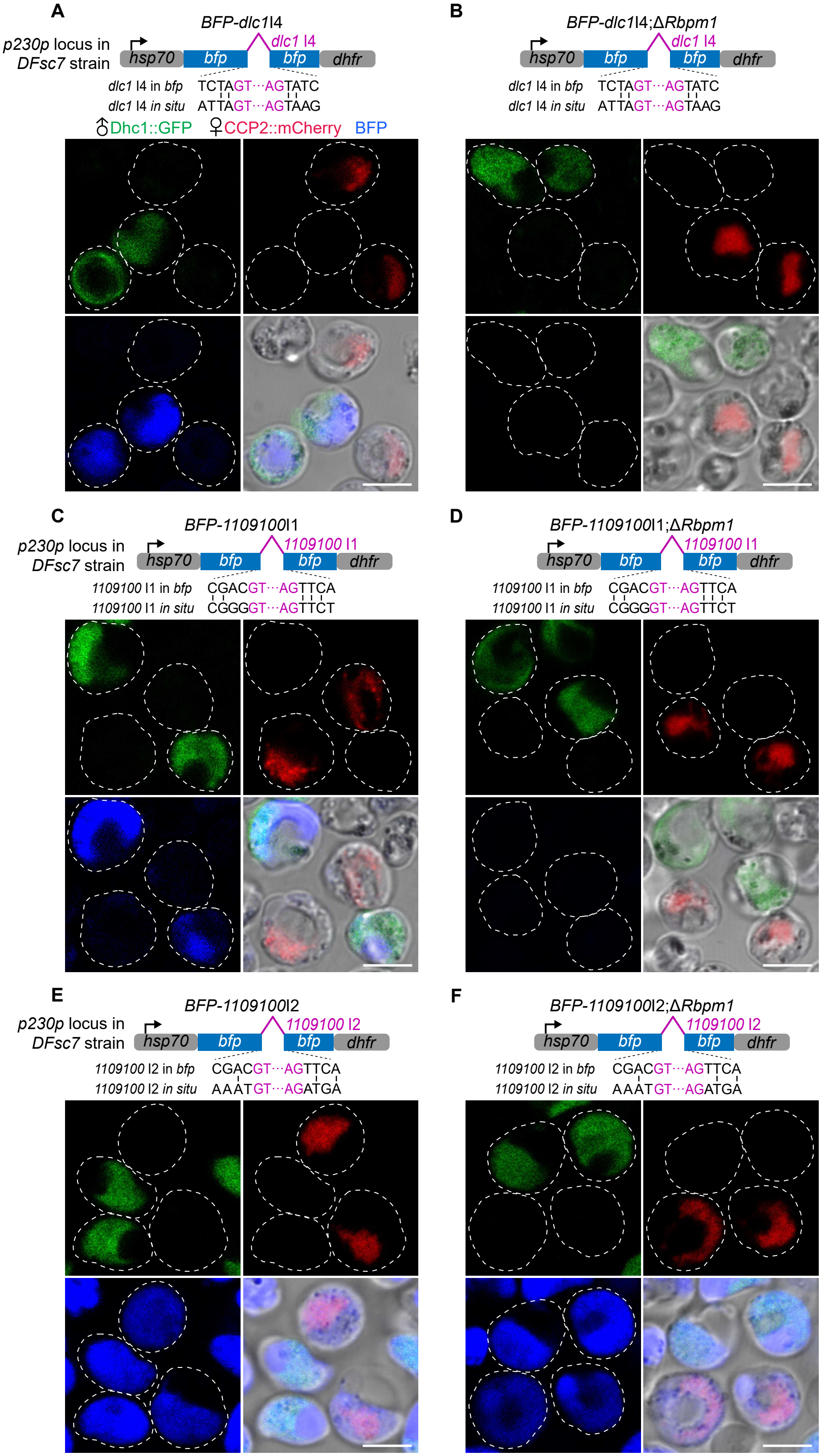

A
BFP-dlc1I4
p230p locus in DFsc7 strain
hsp70
bfp
dlc1 I4
bfp
dhfr
dlc1 I4 in bfp TCTAGT···AGTATC
dlc1 I4 in situ ATTAGT···AGTAAG
♂Dhc1::GFP
♀CCP2::mCherry
BFP
B
BFP-dlc1I4;ΔRbpm1
hsp70
bfp
dlc1 I4
bfp
dhfr
dlc1 I4 in bfp TCTAGT···AGTATC
dlc1 I4 in situ ATTAGT···AGTAAG
C
BFP-1109100I1
p230p locus in DFsc7 strain
hsp70
bfp
1109100 I1
bfp
dhfr
1109100 I1 in bfp CGACGT···AGTTCA
1109100 I1 in situ CGGGGT···AGTTCT
D
BFP-1109100I1;ΔRbpm1
hsp70
bfp
1109100 I1
bfp
dhfr
1109100 I1 in bfp CGACGT···AGTTCA
1109100 I1 in situ CGGGGT···AGTTCT
E
BFP-1109100I2
p230p locus in DFsc7 strain
hsp70
bfp
1109100 I2
bfp
dhfr
1109100 I2 in bfp CGACGT···AGTTCA
1109100 I2 in situ AAATGT···AGATGA
F
BFP-1109100I2;ΔRbpm1
hsp70
bfp
1109100 I2
bfp
dhfr
1109100 I2 in bfp CGACGT···AGTTCA
1109100 I2 in situ AAATGT···AGATGA

Supplementary Figure 10. RBPm1-dependent splicing of axonemal introns inserted in the reporter gene. Related to Figure 7

A. A transgenic line *BFP-dlc1*I4 with a *dlc1* intron4 (*dlc1*I4, purple line)-inserted *bfp* cassette integrated at the *p230p* locus of the *DFsc7* line. Similar analysis as in **Figure 5B**. *dlc1* intron4 was inserted into the *bfp* gene at the nucleotides 455-456 to mimic the splice site (vertical lines) of *in situ dlc1*I4. BFP expression was detected specifically in male gametocytes of the *BFP-dlc1*I4 parasites. Representative for three independent experiments. Scale bars: 5 µm.

B. A *BFP-dlc1*I4 derived RBPm1 mutant line, *BFP-dlc1*I4;Δ*Rbpm1*, showed no BFP expression in male gametocytes. Representative for three independent experiments. Scale bars: 5 µm.

C. Effect of the *PY17X_1109100* intron1 (*BFP-1109100*I1) insertion on the gametocyte expression of BFP. Similar analysis as in **A**. *PY17X_1109100* intron1 was inserted into the *bfp* gene at the nucleotides 390-391 to mimic the splice site (vertical lines) of *in situ PY17X_1109100* intron1. BFP expression was detected specifically in male gametocytes of the *BFP-1109100*I1 parasites.

D. A *BFP-1109100*I1 derived RBPm1 mutant line, *BFP-1109100*I1;Δ*Rbpm1*, showed no BFP expression in male gametocytes. Similar analysis as in **B**.

E. Effect of the *PY17X_1109100* intron2 (*BFP-1109100*I2) insertion on the gametocyte expression of BFP. Similar analysis as in **A**. *PY17X_1109100* intron2 was inserted into the *bfp* gene at the nucleotides 384-385. BFP expression was detected in both male and female gametocytes of the *BFP-1109100*I2 parasites.

F. A *BFP-1109100*I2 derived RBPm1 mutant line *BFP-1109100*I2;Δ*Rbpm1*, showed BFP expression in both male and female gametocytes. Similar analysis as in **B**.

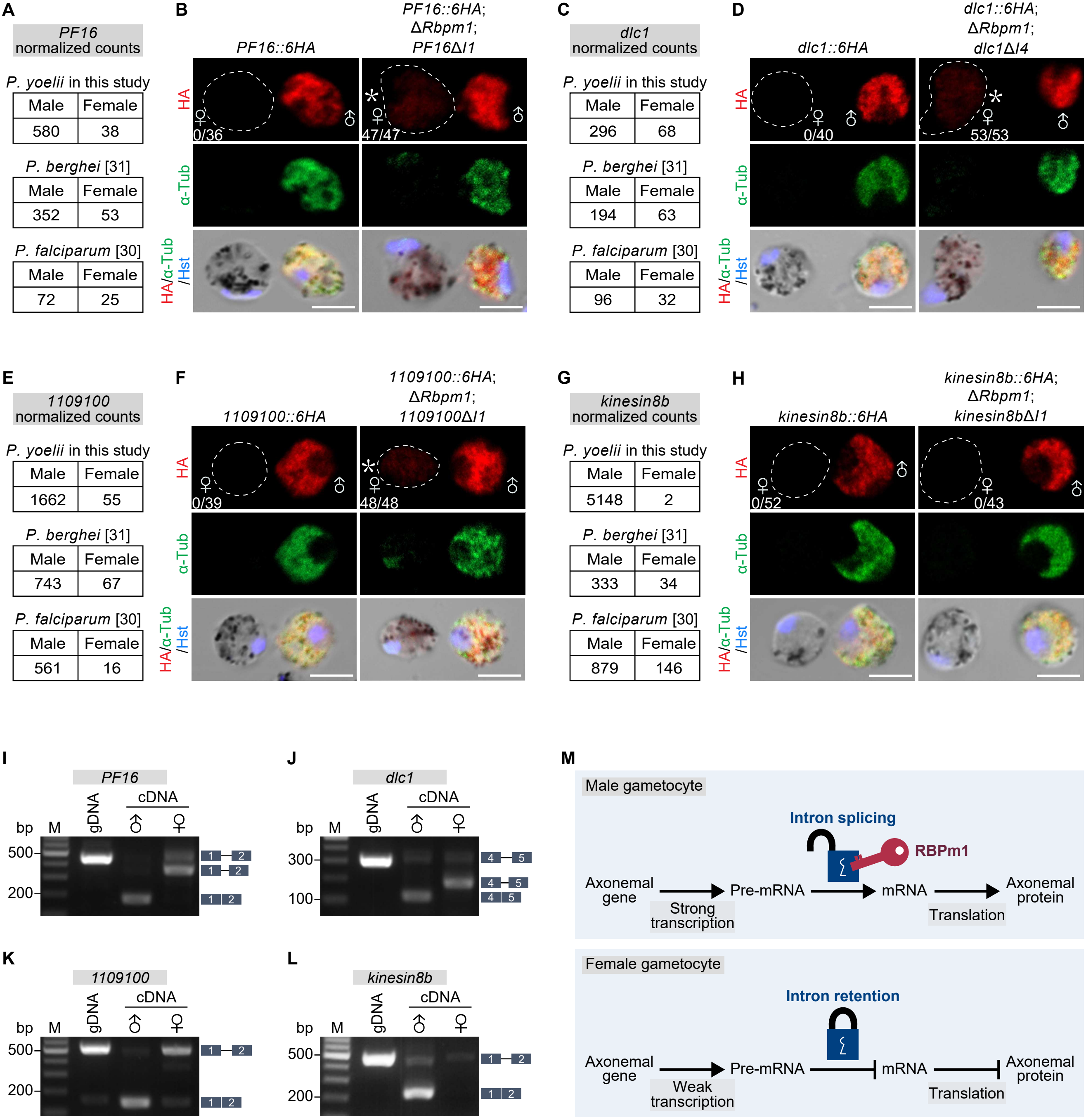
A
PF16 normalized counts
P. yoelii in this study
Male Female
580 38
P. berghei [31]
Male Female
352 53
P. falciparum [30]
Male Female
72 25
B
PF16::6HA
PF16::6HA; ΔRbpm1; PF16ΔI1
HA
α-Tub
HA/α-Tub/Hst
0/36
47/47
C
dlc1 normalized counts
P. yoelii in this study
Male Female
296 68
P. berghei [31]
Male Female
194 63
P. falciparum [30]
Male Female
96 32
D
dlc1::6HA
dlc1::6HA; ΔRbpm1; dlc1ΔI4
0/40
53/53
E
1109100 normalized counts
P. yoelii in this study
Male Female
1662 55
P. berghei [31]
Male Female
743 67
P. falciparum [30]
Male Female
561 16
F
1109100::6HA
1109100::6HA; ΔRbpm1; 1109100ΔI1
0/39
48/48
G
kinesin8b normalized counts
P. yoelii in this study
Male Female
5148 2
P. berghei [31]
Male Female
333 34
P. falciparum [30]
Male Female
879 146
H
kinesin8b::6HA
kinesin8b::6HA; ΔRbpm1; kinesin8bΔI1
0/52
0/43
I
PF16
gDNA
cDNA
bp M
500
200
J
dlc1
300
100
K
1109100
500
200
L
kinesin8b
500
200
M
Male gametocyte
Intron splicing
RBPm1
Axonemal gene
Strong transcription
Pre-mRNA
mRNA
Translation
Axonemal protein
Female gametocyte
Intron retention
Axonemal gene
Weak transcription
Pre-mRNA
mRNA
Translation
Axonemal protein

Supplementary Figure 11. Intron retention prevents expression of axonemal proteins in female gametocytes

A. Transcription level of the *PF16* gene in male and female gametocytes. TMM normalized counts are from the gametocyte transcriptomes in this study and the published dataset contributed by Yeoh, L.M. and Lasonder, E.

B. IFA of 6HA-tagged PF16 in female and male gametocytes of the *PF16::6HA* and *PF16::6HA*;Δ*Rbpm1*;*PF16*Δ*I1* (*PF16* intron1-deleted line described in the **Figure 5D**) parasites. In each image, one male and one female gametocytes were shown. The HA-positive female gametocyte was highlighted with an asterisk. x/y represents the number of HA-positive female gametocytes/the total number of female gametocytes tested. Representative for three independent experiments. Scale bars: 5 μm.

C and D. Low-level expression of the Dlc1 protein detected in female gametocytes after deletion of the *dlc1* intron4. Similar illustration as in **A** and **B**. The *dlc1* intron4-deleted line was described in the **Figure 5G**.

E and F. Low-level expression of the PY17X_1109100 protein detected in female gametocytes after deletion of the *PY17X_1109100* intron1. Similar illustration as in **A** and **B**. The *PY17X_1109100* intron1-deleted line was described in the **Figure 5J**.

G and H. Undetectable expression of the Kinesin8B protein in female gametocytes after deletion of the *kinesin8b* Δ*intron1*. Similar illustration as in **A** and **B**. Note that the *kinesin8b* transcripts are almost undetectable in female gametocytes of *P. yoelii*.

I, J, K, and L. RT-PCR confirmation of intron retention at the transcripts of 4 genes (*PF16*, *dlc1*, *PY17X_1109100*, and *kinesin8b*) in female gametocytes. Genomic DNA (gDNA) from 17XNL parasite, complementary DNA (cDNA) from the purified male and female gametocytes of *DFsc7* were analyzed. Exons are indicated by boxes and introns by lines. Representative for three independent experiments.

M. Proposed different roles of RBPm1-target introns in axonemal gene expression at male and female gametocytes respectively. In male gametocytes, RBPm1 (as a key)-directed splicing of axonemal intron (as a lock) allows protein expression of the axonemal genes. In female gametocytes, dual blockage via weak transcription and IR prevents protein expression of the axonemal genes.

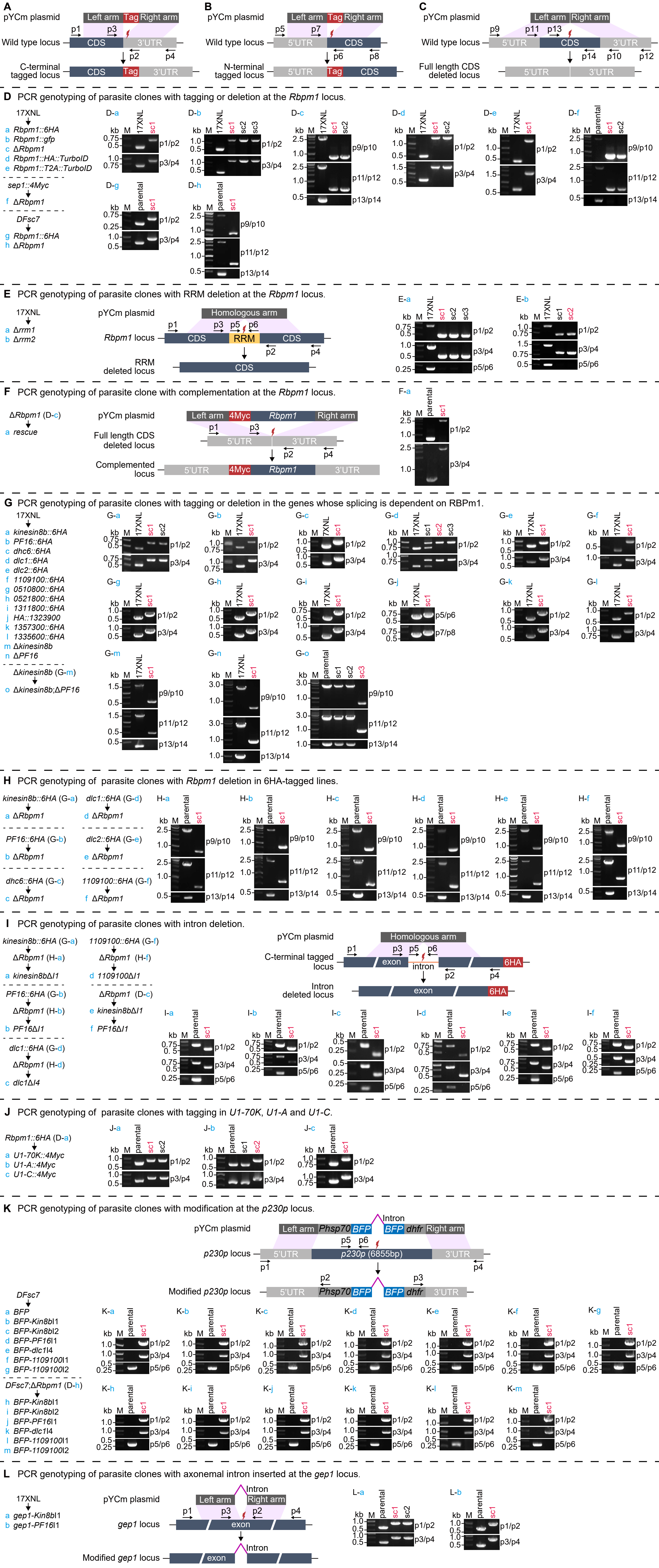

A
pYCm plasmid
Left arm
Tag
Right arm
p1
p3
Wild type locus
CDS
3'UTR
p2
p4
C-terminal tagged locus
CDS
Tag
3'UTR
B
pYCm plasmid
Left arm
Tag
Right arm
p5
p7
Wild type locus
5'UTR
CDS
p6
p8
N-terminal tagged locus
5'UTR
Tag
CDS
C
pYCm plasmid
Left arm
Right arm
p9
p11
p13
Wild type locus
5'UTR
CDS
3'UTR
p14
p10
p12
Full length CDS deleted locus
5'UTR
3'UTR
D PCR genotyping of parasite clones with tagging or deletion at the Rbpm1 locus.
17XNL
a Rbpm1::6HA
b Rbpm1::gfp
c ΔRbpm1
d Rbpm1::HA::TurboID
e Rbpm1::T2A::TurboID
sep1::4Myc
f ΔRbpm1
DFsc7
g Rbpm1::6HA
h ΔRbpm1
D-a
D-b
D-c
D-d
D-e
D-f
D-g
D-h
kb
M
17XNL
parental
sc1
sc2
sc3
p1/p2
p3/p4
p9/p10
p11/p12
p13/p14
E PCR genotyping of parasite clones with RRM deletion at the Rbpm1 locus.
17XNL
a Δrrm1
b Δrrm2
pYCm plasmid
Homologous arm
p1
p3
p5
p6
Rbpm1 locus
CDS
RRM
CDS
p2
p4
RRM deleted locus
CDS
E-a
E-b
p1/p2
p3/p4
p5/p6
F PCR genotyping of parasite clone with complementation at the Rbpm1 locus.
ΔRbpm1 (D-c)
a rescue
pYCm plasmid
Left arm
4Myc
Rbpm1
Right arm
p1
p3
Full length CDS deleted locus
5'UTR
3'UTR
p2
p4
Complemented locus
5'UTR
4Myc
Rbpm1
3'UTR
F-a
p1/p2
p3/p4
G PCR genotyping of parasite clones with tagging or deletion in the genes whose splicing is dependent on RBPm1.
17XNL
a kinesin8b::6HA
b PF16::6HA
c dhc6::6HA
d dlc1::6HA
e dlc2::6HA
f 1109100::6HA
g 0510800::6HA
h 0521800::6HA
i 1311800::6HA
j HA::1323900
k 1357300::6HA
l 1335600::6HA
m Δkinesin8b
n ΔPF16
Δkinesin8b (G-m)
o Δkinesin8b;ΔPF16
G-a
G-b
G-c
G-d
G-e
G-f
G-g
G-h
G-i
G-j
G-k
G-l
G-m
G-n
G-o
p1/p2
p3/p4
p5/p6
p7/p8
p9/p10
p11/p12
p13/p14
H PCR genotyping of parasite clones with Rbpm1 deletion in 6HA-tagged lines.
kinesin8b::6HA (G-a)
a ΔRbpm1
dlc1::6HA (G-d)
d ΔRbpm1
PF16::6HA (G-b)
b ΔRbpm1
dlc2::6HA (G-e)
e ΔRbpm1
dhc6::6HA (G-c)
c ΔRbpm1
1109100::6HA (G-f)
f ΔRbpm1
H-a
H-b
H-c
H-d
H-e
H-f
p9/p10
p11/p12
p13/p14
I PCR genotyping of parasite clones with intron deletion.
kinesin8b::6HA (G-a)
ΔRbpm1 (H-a)
a kinesin8bΔI1
1109100::6HA (G-f)
ΔRbpm1 (H-f)
d 1109100ΔI1
PF16::6HA (G-b)
ΔRbpm1 (H-b)
b PF16ΔI1
ΔRbpm1 (D-c)
e kinesin8bΔI1
f PF16ΔI1
dlc1::6HA (G-d)
ΔRbpm1 (H-d)
c dlc1ΔI4
pYCm plasmid
Homologous arm
C-terminal tagged locus
p1
p3
p5
p6
exon
intron
6HA
p2
p4
Intron deleted locus
exon
6HA
I-a
I-b
I-c
I-d
I-e
I-f
p1/p2
p3/p4
p5/p6
J PCR genotyping of parasite clones with tagging in U1-70K, U1-A and U1-C.
Rbpm1::6HA (D-a)
a U1-70K::4Myc
b U1-A::4Myc
c U1-C::4Myc
J-a
J-b
J-c
p1/p2
p3/p4
K PCR genotyping of parasite clones with modification at the p230p locus.
pYCm plasmid
Left arm
Phsp70
BFP
Intron
BFP
dhfr
Right arm
p5
p6
p230p locus
5'UTR
p230p (6855bp)
3'UTR
p1
p4
p2
p3
Modified p230p locus
5'UTR
Phsp70
BFP
BFP
dhfr
3'UTR
DFsc7
a BFP
b BFP-Kin8bI1
c BFP-Kin8bI2
d BFP-PF16I1
e BFP-dlc1I4
f BFP-1109100I1
g BFP-1109100I2
DFsc7;ΔRbpm1 (D-h)
h BFP-Kin8bI1
i BFP-Kin8bI2
j BFP-PF16I1
k BFP-dlc1I4
l BFP-1109100I1
m BFP-1109100I2
K-a
K-b
K-c
K-d
K-e
K-f
K-g
K-h
K-i
K-j
K-k
K-l
K-m
p1/p2
p3/p4
p5/p6
L PCR genotyping of parasite clones with axonemal intron inserted at the gep1 locus.
17XNL
a gep1-Kin8bI1
b gep1-PF16I1
pYCm plasmid
Left arm
Intron
Right arm
p1
p3
p2
p4
gep1 locus
exon
Modified gep1 locus
exon
Intron
L-a
L-b
p1/p2
p3/p4

Supplementary Figure 12. Genotyping of genetically modified parasite lines

A-C. Schematic diagrams showing CRISPR/Cas9-mediated gene tagging at C-terminus (**A**), gene tagging at N-terminus (**B**), and gene deletion (**C**) via double cross-over homologous recombination. 'p' represents the primers for PCR, and the red lightning bolt represents the sites for sgRNA recognition.

D-L. Genotyping PCR results showing correct 5' and 3' homologous recombination in the modified parasite lines in this study. For each modification, usually 1-3 parasite single clones (sc) were obtained via limiting dilution. The red-colored sc was selected for further analysis. Oligo sequences used for plasmid construction and primers used for genotyping PCR are listed in Supplementary Table 2.

A

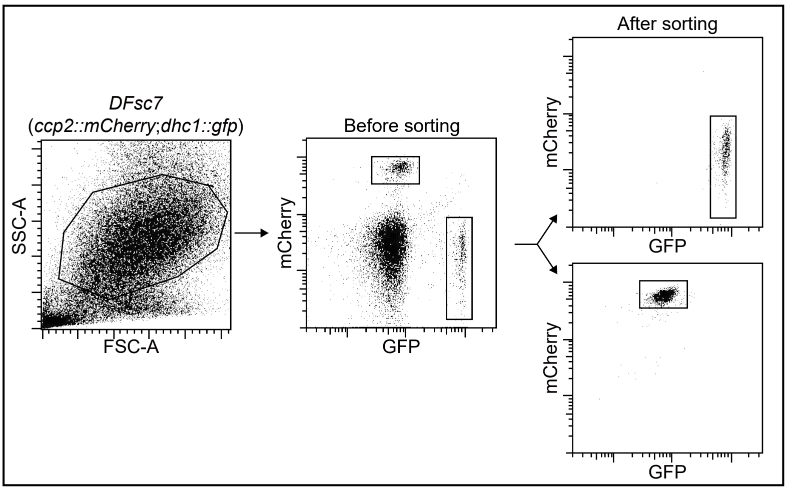

DFsc7
(*ccp2::mCherry*;*dhc1::gfp*)
SSC-A
FSC-A
Before sorting
mCherry
GFP
After sorting
mCherry
GFP
mCherry
GFP

B

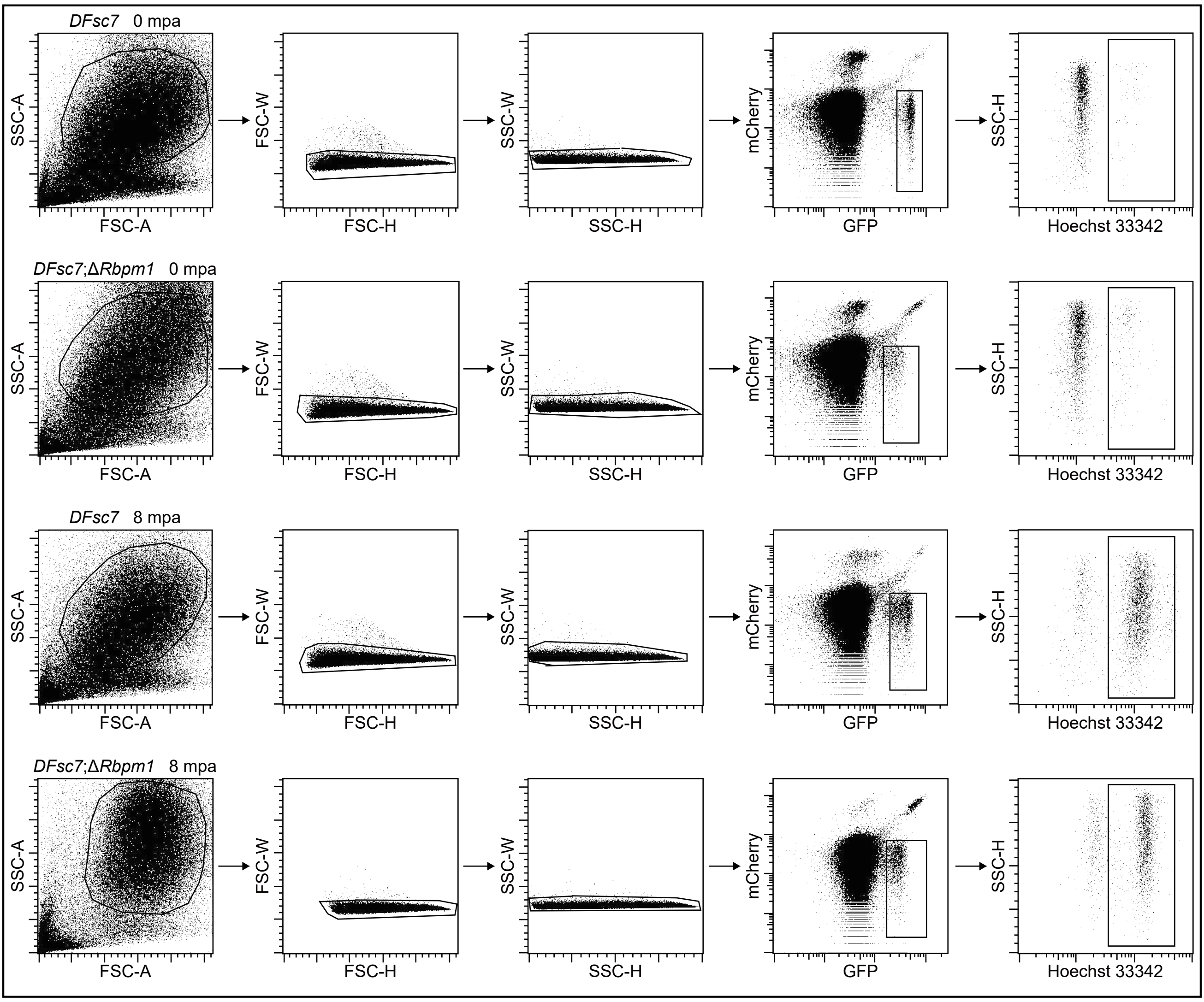

DFsc7 0 mpa
DFsc7;Δ*Rbpm1* 0 mpa
DFsc7 8 mpa
DFsc7;Δ*Rbpm1* 8 mpa
SSC-A
FSC-A
FSC-W
FSC-H
SSC-W
SSC-H
mCherry
GFP
SSC-H
Hoechst 33342

C

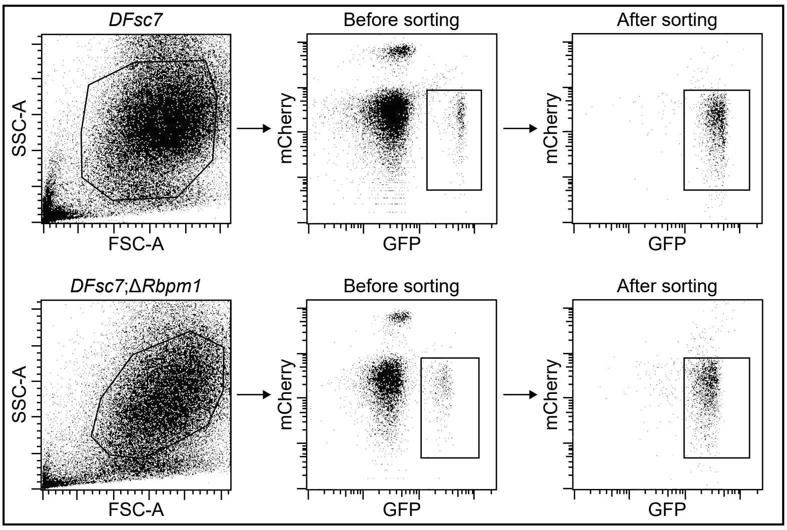

DFsc7
DFsc7;Δ*Rbpm1*
SSC-A
FSC-A
Before sorting
After sorting
mCherry
GFP

Supplementary Figure 13. Flow cytometry gating strategies

A. Gating strategy for sorting male and female gametocytes from a *P. yoelii* parasite reporter line *DFsc7* presented in Supplementary Fig. 1A. Forward and side scatter signals were used to distinguish red blood cells from debris, doublets, and white blood cells. Male and female gametocytes were sorted based on GFP fluorescence and mCherry fluorescence. After sorting, gametocyte purity was assessed by re-analysis of a sample fraction.

B. Gating strategy for analyzing DNA content of male gametocytes presented in Supplementary Fig. 2A. Forward and side scatter signals were used to distinguish red blood cells from debris, doublets, and white blood cells. Male gametocytes were identified by GFP fluorescence and analyzed for Hoechst 33342 fluorescence.

C. Gating strategy for sorting male gametocytes from the *DFsc7* and *DFsc7*;Δ*Rbpm1* lines presented in Supplementary Fig. 3F. Forward and side scatter signals were used to distinguish red blood cells from debris, doublets, and white blood cells. Male gametocytes were sorted based on GFP fluorescence. After sorting, male gametocyte purity was assessed by re-analysis of a sample fraction.

Table S1. List of genetically modified parasite strains in this study

Strain	Parental strain	Description	Resource
17XNL	/	*Plasmodium yoelii* 17XNL strain	NIH
**Parasites with gene tagging**			
DFsc7	17XNL	*DFsc7* expresses mCherry and GFP reporters mutual-exclusively in the female and male gametocytes	Liu et al. 2018
sep1::4Myc	17XNL	*sep1* C-terminally tagged with 4Myc	Jiang et al. 2020
Rbpm1::6HA	17XNL	*Rbpm1* C-terminally tagged with 6HA	Fig S12
Rbpm1::gfp	17XNL	*Rbpm1* C-terminally tagged with GFP	Fig S12
DFsc7 ;Rbpm1::6HA	*DFsc7*	*Rbpm1* C-terminally tagged with 6HA	Fig S12
Rbpm1::HA::TurboID	17XNL	*Rbpm1* C-terminally tagged with HA-TurboID	Fig S12
Rbpm1::T2A::TurboID	17XNL	*Rbpm1* C-terminally tagged with 3NLS-HA-TurboID	Fig S12
kinesin8b::6HA	17XNL	*kinesin8b* C-terminally tagged with 6HA	Fig S12
PF16::6HA	17XNL	*PF16* C-terminally tagged with 6HA	Fig S12
dhc6::6HA	17XNL	*dhc6* C-terminally tagged with 6HA	Fig S12
dhc7::6HA	17XNL	*0510800* C-terminally tagged with 6HA	Fig S12
dlc1::6HA	17XNL	*dlc1* C-terminally tagged with 6HA	Fig S12
dlc2::6HA	17XNL	*dlc2* C-terminally tagged with 6HA	Fig S12
1109100::6HA	17XNL	*1109100* C-terminally tagged with 6HA	Fig S12
0521800::6HA	17XNL	*0521800* C-terminally tagged with 6HA	Fig S12
1311800::6HA	17XNL	*1311800* C-terminally tagged with 6HA	Fig S12
HA::1323900	17XNL	*1323900* N-terminally tagged with HA	Fig S12
1357300::6HA	17XNL	*1357300* C-terminally tagged with 6HA	Fig S12
1335600::6HA	17XNL	*1335600* C-terminally tagged with 6HA	Fig S12
Rbpm1::6HA ;U1-70K::4Myc	*Rbpm1::6HA*	*U1-70K* C-terminally tagged with 4Myc	Fig S12
Rbpm1::6HA ;U1-A::4Myc	*Rbpm1::6HA*	*U1-A* C-terminally tagged with 4Myc	Fig S12
Rbpm1::6HA ;U1-C::4Myc	*Rbpm1::6HA*	*U1-C* C-terminally tagged with 4Myc	Fig S12
**Parasites with gene knockout**			
ΔRbpm1	17XNL	Deletion of the whole coding sequences of *Rbpm1*	Fig S12
Δnke4	17XNL	Deletion of the partial coding sequences of *nek4*	Jiang et al. 2020
Δmap2	17XNL	Deletion of the partial coding sequences of *map2*	Jiang et al. 2020
sep1::4Myc ;ΔRbpm1	*sep1::4Myc*	Deletion of the whole coding sequences of *Rbpm1*	Fig S12
DFsc7 ;ΔRbpm1	*DFsc7*	Deletion of the whole coding sequences of *Rbpm1*	Fig S12
Δkinesin8b	17XNL	Deletion of the N-terminal 1184 bp coding sequence of *kinesin8b*	Fig S12
ΔPF16	17XNL	Deletion of the whole coding sequences of *PF16*	Fig S12
Δkinesin8b ;ΔPF16	*Δkinesin8b*	Deletion of the whole coding sequences of *PF16*	Fig S12
kinesin8b::6HA ;ΔRbpm1	*kinesin8b::6HA*	Deletion of the whole coding sequences of *Rbpm1*	Fig S12
PF16::6HA ;ΔRbpm1	*PF16::6HA*	Deletion of the whole coding sequences of *Rbpm1*	Fig S12
dhc6::6HA ;ΔRbpm1	*dhc6::6HA*	Deletion of the whole coding sequences of *Rbpm1*	Fig S12
dlc1::6HA ;ΔRbpm1	*dlc1::ΔHA*	Deletion of the whole coding sequences of *Rbpm1*	Fig S12
dlc2::6HA ;ΔRbpm1	*dlc2::6HA*	Deletion of the whole coding sequences of *Rbpm1*	Fig S12
1109100::6HA ;ΔRbpm1	*1109100::6HA*	Deletion of the whole coding sequences of *Rbpm1*	Fig S12
Δgep1	17XNL	Deletion of the whole coding sequences of *gep1*	Jiang et al. 2020
**Parasites with RRM deletion at the *Rbpm1* locus**			
Δrrm1	17XNL	Deletion of the 565-981 bp coding sequence of *Rbpm1*	Fig S12
Δrrm2	17XNL	Deletion of the 1018-1519 bp coding sequence of *Rbpm1*	Fig S12
**Parasites with gene compelementation**			
rescue	*ΔRbpm1*	*In situ* complementation of *Rbpm1* N-terminally tagged with 4Myc	Fig S12
**Parasites with intron deletion**			
kinesin8b::6HA ;ΔRbpm1 ;kinesin8b ΔI1	*kinesin8b::6HA ;ΔRbpm1*	Deletion of *Kinesin8b* intron 1	Fig S12
PF16::6HA ;ΔRbpm1 ;PF16 ΔI1	*PF16::6HA ;ΔRbpm1*	Deletion of *PF16* intron 1	Fig S12
dlc1::6HA ;ΔRbpm1 ; dlc1 ΔI4	*dlc1::6HA ;ΔRbpm1*	Deletion of *dlc1* intron4	Fig S12
1109100::6HA ;ΔRbpm1 ;1109100 ΔI1	*1109100::6HA ;ΔRbpm1*	Deletion of *1109100* intron 1	Fig S12
ΔRbpm1 ;kinesin8b ΔI1	*ΔRbpm1*	Deletion of *kinesin8b* intron 1	Fig S12
ΔRbpm1 ;kinesin8b ΔI1 ;PF16 ΔI1	*ΔRbpm1 ;kinesin8b ΔI1*	Deletion of *PF16* intron 1	Fig S12
**Parasites with modification at the *p230p* locus**			
BFP	*DFsc7*	Coding region of *p230p* gene was replaced with the *BFP* expression cassette	Fig S12
BFP-Kin8b I1	*DFsc7*	Coding region of *p230p* gene was replaced with the *BFP-Kin8b* I1 expression cassette	Fig S12
BFP-Kin8b I1;*ΔRbpm1*	*DFsc7 ;ΔRbpm1*	Coding region of *p230p* gene was replaced with the *BFP-Kin8b* I1 expression cassette	Fig S12
BFP-Kin8b I2	*DFsc7*	Coding region of *p230p* gene was replaced with the *BFP-Kin8b* I2 expression cassette	Fig S12
BFP-Kin8b I2;*ΔRbpm1*	*DFsc7 ;ΔRbpm1*	Coding region of *p230p* gene was replaced with the *BFP-Kin8b* I2 expression cassette	Fig S12
BFP-PF16 I1	*DFsc7*	Coding region of *p230p* gene was replaced with the *BFP-PF16* I1 expression cassette	Fig S12
BFP-PF16 I1;*ΔRbpm1*	*DFsc7 ;ΔRbpm1*	Coding region of *p230p* gene was replaced with the *BFP-PF16* I1 expression cassette	Fig S12
BFP-dlc1 I4	*DFsc7*	Coding region of *p230p* gene was replaced with the *BFP-dlc1* I4 expression cassette	Fig S12
BFP-dlc1 I4;*ΔRbpm1*	*DFsc7 ;ΔRbpm1*	Coding region of *p230p* gene was replaced with the *BFP-dlc1* I4 expression cassette	Fig S12
BFP-1109100 I1	*DFsc7*	Coding region of *p230p* gene was replaced with the *BFP-1109100* I1 expression cassette	Fig S12
BFP-1109100 I1;*ΔRbpm1*	*DFsc7 ;ΔRbpm1*	Coding region of *p230p* gene was replaced with the *BFP-1109100* I1 expression cassette	Fig S12
BFP-1109100 I2	*DFsc7*	Coding region of *p230p* gene was replaced with the *BFP-1109100* I2 expression cassette	Fig S12
BFP-1109100 I2;*ΔRbpm1*	*DFsc7 ;ΔRbpm1*	Coding region of *p230p* gene was replaced with the *BFP-1109100* I2 expression cassette	Fig S12
**Parasites with axonemal intron inserted at the *gep1* locus**			
gep1-Kin8b I1	17XNL	Insertion of *Kinesin8b* intron 1 into exon 3 of *gep1*	Fig S12
gep1-PF16 I1	17XNL	Insertion of *PF16* intron 1 into exon 1 of *gep1*	Fig S12
nt: not tested			

Table S2. Oligonucleotides and primers used in this study

Oligo sequences for constructing gene tagging plasmids

Gene name	Gene ID	Tag	Left homologous arm		Right homologous arm		Target site of sgRNA	
			Forward primer	Reverse primer	Forward primer	Reverse primer	Forward oligo	Reverse oligo
Rbpm1	PY17X_0716700	C-terminal 6HA	CCCAAGCTTGCCGAACCTAAGAAGCCAAA	CATGCCATGGATTGTTATAATCCGGTTGTTGC	CCGCTCGAGTTTATGTCATTTTTTGAGGT	CCGGAATTCGTTCCAATGAAGACAAACAA	TATTGTAAGGGAAAGGACCCATAAA	AAACTTTATGGGTCCTTTCCCTTAC
Rbpm1	PY17X_0716700	C-terminal GFP	CCCAAGCTTGCCGAACCTAAGAAGCCAAA	CATGCCATGGATTGTTATAATCCGGTTGTTGC	CCGCTCGAGTTTATGTCATTTTTTGAGGT	CCGGAATTCGTTCCAATGAAGACAAACAA	TATTGTAAGGGAAAGGACCCATAAA	AAACTTTATGGGTCCTTTCCCTTAC
Rbpm1	PY17X_0716700	C-terminal HA-TurboID	CCCAAGCTTGCCGAACCTAAGAAGCCAAA	CATGCCATGGATTGTTATAATCCGGTTGTTGC	CCGCTCGAGTTTATGTCATTTTTTGAGGT	CCGGAATTCGTTCCAATGAAGACAAACAA	TATTGTAAGGGAAAGGACCCATAAA	AAACTTTATGGGTCCTTTCCCTTAC
Rbpm1	PY17X_0716700	C-terminal 3NLS-HA-TurboID	CCCAAGCTTGCCGAACCTAAGAAGCCAAA	CATGCCATGGATTGTTATAATCCGGTTGTTGC	CCGCTCGAGTTTATGTCATTTTTTGAGGT	CCGGAATTCGTTCCAATGAAGACAAACAA	TATTGTAAGGGAAAGGACCCATAAA	AAACTTTATGGGTCCTTTCCCTTAC
kinesin8b	PY17X_0204100	C-terminal 6HA	CCCAAGCTTGATGACCAAATGAAGAGCTT	CATGCCATGGAGTTTTATTTTTTGTGATGCTAG	CCGCTCGAGAAATGTTTGAACTTTACCATTC	GGGCTTAAGGAAAAAGGCAATGATGCCAT	TATTGTGCGTCAGTTAATTTATGA	AAACTCATAAATTAACTGACGCAC
PF16	PY17X_0919000	C-terminal 6HA	CCCAAGCTTACATCCTGTATTTCTAAAATC	CATGCCATGGTTCTTCGGTATTTTCAACTTC	CCGCTCGAGAACAAGGAAATAATAACAAGT	GGGCTTAAGCTCGCAATTCCTCCTTTTGC	TATTGTGTGTACATACTTCATGTG	AAACCACATGAAGTATGTACACAC
dhc6	PY17X_0603800	C-terminal 6HA	CCCAAGCTTGTGTGTGCAAACGCAAATAT	CATGCCATGGATTATATATAAATAAATAGA	CCGCTCGAGAAAAAATACGATATACAAAT	CCGGAATTCACGATTATATTATACCAGTA	TATTGGAAGTAGAAATGCCGTTGT	AAACACAACGGCATTTCTACTTCC
dhc7	PY17X_0510800	C-terminal 6HA	CGGGGTACCTGATGATAATGAATTTATTT	CATGCCATGGTAGTAGCATATGAACTTTAA	CCGCTCGAGTTTTTCGTTTAATTTTTTAA	CCCCTTAAGCATCACATTCTTTCATTTTA	TATTGTGGAGACTAGGATCTGAAGG	AAACCCTTCAGATCCTAGTCTCCAC
dlc1	PY17X_1241500	C-terminal 6HA	CCCAAGCTTCTCGAAAATGCTTCCTCTCAA	CATGCCATGGAAGATGAGCTACGGATCCATC	CCGCTCGAGATTGAAAAGGCAACATGCTCA	CCGGAATTCCAAAATTATAGGAAATTGCAA	TATTGTTACTGAAAAGTACGTTATA	AAACTATAACGTACTTTTCAGTAAC
dlc2	PY17X_0302800	C-terminal 6HA	CGGGGTACCCCTTATCATAATCAGAATAA	CATGCCATGGACACTTATATACAACAATTG	CCGCTCGAGAATATGATAATTAAATGTGG	GGGCTTAAGGGGGTGAAAAAATAATTTGG	TATTGCTATATGATGTATGCTATA	AAACTATAGCATACATCATATAGC
1109100	PY17X_1109100	C-terminal 6HA	CCCAAGCTTGTACCACAAACAATGGTGGA	CGGGGTACCGGATTGTCGTCATTATTGTAAGTG	CCGCTCGAGGGGGTCCATATAAGATGGAA	CCGGAATTCTGGGTTTAACCTTGTACACC	TATTGTTGCTGCTTGGCTTGTTGCT	AAACAGCAACAAGCCAAGCAGCAAC
0521800	PY17X_0521800	C-terminal 6HA	CGGGGTACCCAATAAATAGTGGAAACAAA	CATGCCATGGCAGTTTATCCCCTAATAAAT	CCGCTCGAGATATTGATAATAATAATCTC	CCCCTTAAGGGGAAACAATATATACGAAT	TATTGTGAAGTCTATATGACCAAGT	AAACACTTGGTCATATAGACTTCAC
1311800	PY17X_1311800	C-terminal 6HA	CGGGGTACCCCCCGAGGACCAATCTTTTG	CATGCCATGGTTGTCCAGTTAAATAATACA	CCGCTCGAGAAAAAAAGAGAAATAAAGAA	CCCCTTAAGGTGTGCCTAAAAGTATGTTC	TATTAATCAGAGGATGAAGTTGCA	AAACTGCAACTTCATCCTCTGATT
1323900	PY17X_1323900	N-terminal HA	CCCAAGCTTGGCATTACTGTCGAAGGACT	CATGCCATGGATTAATTACAAGGAAAGGTA	CCGCTCGAGATGGAACAAAAAAAAAATAG	CCCCTTAAGACCTGTTTATCAAAATGCGA	TATTGTAAATATGAAGGGAGGAGGA	AAACTCCTCCTCCCTTCATATTTAC
1357300	PY17X_1357300	C-terminal 6HA	CGGGGTACCACCCCCGATCTTAATACAAC	CATGCCATGGATCTATTTCAAAATAATTTT	CCGCTCGAGCATACAAATAAATTAAAAAG	CCCCTTAAGTATGCCAGCTTGTATGCCAG	TATTGCATATGCACATTATCAAAAG	AAACCTTTTGATAATGTGCATATGC
1335600	PY17X_1335600	C-terminal 6HA	CCCAAGCTTCGAAATAAATGGAGATAAA	CATGCCATGGCTTTTTTTTATATTTGTCTTTAAAAG	CCGCTCGAGAATTTTTATAATGAACTTAGC	CCGGAATTCCTAATGATCTGTTAATTGC	TATTGCCCTTTATGGCTACCTATTA	AAACTAATAGGTAGCCATAAAGGGC
U1-70K	PY17X_1144300	C-terminal 4Myc	CCCAAGCTTGGAAAAGGTCCTTCTAGGGG	CATGCCATGGTTCATACCCTTCATTATGATC	CCGCTCGAGAAGTTTTTCGTAAAAAATATCATAAT	CCGGAATTCGTAACTTGCTTAAAATGACA	TATTGACAGAGATAGAGGCAGAGA	AAACTCTCTGCCTCTATCTCTGTC
U1-A	PY17X_1407100	C-terminal 4Myc	CCCAAGCTTCAGGATTTGTTGAAGCCAGA	CATGCCATGGTCGTTTCGCATATGATATTTTTAAC	CCGCTCGAGCCTTTTTTTTTTTTTTGGAATTATT	GGGCTTAAGGCATATCTGTGTGCTTTGTA	TATTGTATGTCCATATTCCCTATTT	AAACAAATAGGGAATATGGACATAC
U1-C	PY17X_1426800	C-terminal 4Myc	CCCAAGCTTCACATAGTTCACCAGTAGGT	CATGCCATGGCTCATTATTTTTATTTACAAAATTTGC	CCGCTCGAGCCCAATTAAGGGAAGTATAT	GGGCTTAAGGAGTATATACACGTTCGAAA	TATTAGAGCAGGCTATGTTCCCCC	AAACGGGGGAACATAGCCTGCTCT

Diagnositc PCR primers for C-terminal tagging

Gene name	Gene ID	Tag	P1	P2	P3	P4		
Rbpm1	PY17X_0716700	C-terminal 6HA	GGATAAAATGGTTGAAGTTAG	GGTTAAAAGCTAAAAAGGCC	TGAAAATATGCAACAACCGG	TGATTCTACCTGTACACCAG		
Rbpm1	PY17X_0716700	C-terminal GFP	GGATAAAATGGTTGAAGTTAG	GGTTAAAAGCTAAAAAGGCC	TGAAAATATGCAACAACCGG	TGATTCTACCTGTACACCAG		
Rbpm1	PY17X_0716700	C-terminal HA-TurboID	GGATAAAATGGTTGAAGTTAG	GGTTAAAAGCTAAAAAGGCC	TGAAAATATGCAACAACCGG	TGATTCTACCTGTACACCAG		
Rbpm1	PY17X_0716700	C-terminal 3NLS-HA-TurboID	GGATAAAATGGTTGAAGTTAG	GGTTAAAAGCTAAAAAGGCC	TGAAAATATGCAACAACCGG	TGATTCTACCTGTACACCAG		
Rbpm1	PY17X_0716700	C-terminal 6HA	GGATAAAATGGTTGAAGTTAG	GGTTAAAAGCTAAAAAGGCC	TGAAAATATGCAACAACCGG	TGATTCTACCTGTACACCAG		
kinesin8b	PY17X_0204100	C-terminal 6HA	TTAATAAACAAACTAGTTGC	GAATGGTAAAGTTCAAACATTT	CGCACCAAAGATCTCAAACC	CCCACTTAGATATTTCGAAA		
PF16	PY17X_0919000	C-terminal 6HA	CTAATGAGTCATCTGATGAT	TATAGTAAGAAATAGCAGAC	CACACCTGGATATTCTGAAA	CCTTGAAGTAATCTTATCAC		
dhc6	PY17X_0603800	C-terminal 6HA	CGTTAAGATATGCAACTGTC	TACAAAAATAGCAAACGGTT	CAAATCTGAAAAACATAAATCC	GTCTCGAACAAATTTAAATG		
dhc7	PY17X_0510800	C-terminal 6HA	GACGATGAATCAAATTCGAA	ACAGGTTAAAAAATTAAACG	GCCATGCATCCATAGCAAAA	GTTTTGAATTTCTTCTAACTC		
dlc1	PY17X_1241500	C-terminal 6HA	GGTCCAACATAATTTGTGAAG	GAAAATTTAGAAAACATATGT	AGATGGATCCGTAGCTCATC	GACCTCTTACTATGTGTAAC		
dlc2	PY17X_0302800	C-terminal 6HA	GGCATAAGTTGAATAAGCATTCA	CATATGAGTGTTTTCATCCC	GCATTATTTTGTAGGAGGAGT	ATCATTCCCTATATTTCCTGAATT		
1109100	PY17X_1109100	C-terminal 6HA	TCTACAAAAGAGGAAGCTTC	TTCCATCTTATATGGACCCC	GACACTTACAATAATGACGA	CCGAAGCACAATTAGCAATC		
0521800	PY17X_0521800	C-terminal 6HA	CAAGGTAATAATATGTACATG	AACTTCCATGATATTTTGAG	ATTTATTAGGGGATAAACTG	TGTGTATATTGACAACATCC		
1311800	PY17X_1311800	C-terminal 6HA	ATAGGAAAAACTGGATTTCC	TCCCTTTGTTTTTTTCGTTG	CAGAGGATGAAGTTGCATGG	GGCGTAAGCCAAAGAATGAAC		
1357300	PY17X_1357300	C-terminal 6HA	AAAACTGCAGGAGAAAATGC	CGGGGGAAGGGGTATATTTG	CAACTTCCGATAATTCAATAG	ACAATATGCCACGTGCACAA		
1335600	PY17X_1335600	C-terminal 6HA	CTGATGATATTTTTGAAACC	TAGGAAATATGAATTAGGAG	AAAATCGAGAGATGCAAAAC	ATGATTATGGGGATATAGTC		
U1-70K	PY17X_1144300	C-terminal 4Myc	TACCTAGAAGATTAGGAGGG	TATCATTTGGAATATCAAAGG	GCCAGGAAAATGGAGAATAT	CCCTATATATGTGGTTATATC		
U1-A	PY17X_1407100	C-terminal 4Myc	GAAAATGTTAATACTGAAGC	CTACATATATTGTGCGTTTT	AGTTAAAAATATCATATGCG	TAACGGATGGTTAGATAAGA		
U1-C	PY17X_1426800	C-terminal 4Myc	GTGAATATTGCGATATATATC	ATAACTTAAACATTTCATGTG	CAAATGAATAAAGAAAATGC	CGGAATAACTGCTCATTTAT		

Diagnositc PCR primers for N-terminal tagging

Gene name	Gene ID	Tag	P5	P6	P7	P8		
1323900	PY17X_1323900	N-terminal HA	GGGGGGAATGATGGAAAAAA	ACTAAATGAAGGCGACTCAC	GTTCTTTTACCTTTCCTTGT	GGAAAGGTTTTGTGTAGCTA		
kinesin8b	PY17X_0204100	N-terminal 6HA	GTTTACTCTCTTGTCCACAT	GATCCTGCACATTTTTAAGA	GTGGATCTACTTGATATACT	CATCATAATATTGAGTTTTA		
PF16	PY17X_0919000	N-terminal 6HA	GCAAAAATGTAGACAATAATCC	CGACCTATTATAATCGTCAA	AAAAAATTTCACAAAAAAGG	GGTATGATACCTTTATCAAC		

Oligo sequences for constructing gene knockout plasmids

Gene name	Gene ID	Gene size (bp) / deleted gene size (bp)	Left homologous arm		Right homologous arm		Target site of sgRNA	
			Forward primer	Reverse primer	Forward primer	Reverse primer	Forward oligo	Reverse oligo
Rbpm1	PY17X_0716700	1904/1904	CGGGGTACCGCATACACGAGGAAATACTA	CATGCCATGGTTTGTGTATTTTATTTTTCGTCG	CCGCTCGAGTTTATGTCATTTTTTGAGGT	CCGGAATTCGTTCCAATGAAGACAAACAA	TATTGTAGGGGGGTTAAGCTACTAT	AAACATAGTAGCTTAACCCCCCTAC
kinesin8b	PY17X_0204100	5137/1184	CGGGGTACCCCGGTTGTGCATTTATTATT	CATGCCATGGATTTTGATCTTTACTATATT	CCGCTCGAGACAGAACAACTTAATGGACAG	CCGGAATTCCTTTCTCTTCATTCATTTCTTG	TATTGTTACCCTCAGTATACAAAAT	AAACATTTTGTATACTGAGGGTAAC
PF16	PY17X_0919000	1809/1809	CCCAAGCTTACGGTTTCTAAAGTAATAATATCAC	CATGCCATGGTTTTTTTTTTTTTTATTAACAGTCA	CCGCTCGAGAACAAGGAAATAATAACAAGT	GGGCTTAAGCTCGCAATTCCTCCTTTTGC	TATTAGTGCATGTGTTTGGACTGT	AAACACAGTCCAAACACATGCACT

Diagnostic PCR primers for gene knockout

Gene name	Gene ID	P9	P10	P11	P12	P13	P14	
Rbpm1	PY17X_0716700	AAGCGAAATAAATGAAACGG	GGTTAAAAGCTAAAAAGGCC	CGACGAAAAATAAAATACACAAA	CGTAATTTCAAAAATGAAGA	GCGGACACTCTAAGTACTGG	GGTTACAAAGGCAAACCCTC	
kinesin8b	PY17X_0204100	GTTTACTCTCTTGTCCACAT	CTGTCCATTAAGTTGTTCTGT	CTATATATGTGGATCTACTTG	TATGATATTTTTGGCGTCTC	CGATGCAAACTATAACATCT	GATTCTGTATGTTGAGGGGT	
PF16	PY17X_0919000	CAGATTATTGTCATATCATC	AAAGCACAATAATGTATTGG	CCTCCCCACTTTTAGTACTG	CGACATGACTTGTACAGGTG	GCAACAGTAATACTAGCGAA	AAGCTTCAAGATCCGTAACT	

Oligo sequences for constructing RRM deletion plasmids

Strain	Homologous arm		Mutation primers		Target site of sgRNA	
	Forward primer	Reverse primer	Forward primer	Reverse primer	Forward oligo	Reverse oligo
Δrrm1	CCGCTCGAGCGAGAACGAAGTTTATCTAA	GGGCTTAAGGGACATATACATATTCTCCT	GTAACTCAAAAAATATAAATGAACCATTTGAGGAACACAATACG	ATCTGAGGGTGTCGTATTGTGTTCCTCAAATGGTTCATTTATAT	TATTATTAGAAAAATAAAAATTGT	AAACACAATTTTTATTTTTCTAAT
Δrrm2	CCGCTCGAGCATTGGAGGAATTCCACAAA	GGGCTTAAGTGTTATAATCCGGTTGTTGC	CGACACCCTCAGATATTCAAAGAAAAGCCGAACCTAAG	TTGGCTTCTTAGGTTCGGCTTTTCTTTGAATATCTGAG	TATTGTAAGGGAAAGGACCCATAAA	AAACTTTATGGGTCCTTTCCCTTAC

Diagnostic PCR primers for RRM deletion

Strain	P1	P2	P3	P4	P5	P6		
Δrrm1	CAACAATTGTGAAAACAGAG	AGGGTGTCGTATTGTGTTCC	CTTCAACAGGAATTATTGAA	CTCCTCATTCCACTATTATT	TTTCATTGGAGGAATTCCAC	ATTTATATGAGATGACATGG		
Δrrm2	GTTGGCAACATATCTGAACT	ATGAGCCAATACATACACAC	ATTGAATGGGAAACGAGTGG	GTTCCAATGAAGACAAACAA	GGGGGTTAAGCTACTATTGG	CTAACTTCAACCATTTTATC		

Oligo sequences for constructing gene *in situ* complementation plasmids

Gene name	Gene ID	Tag	Left homologous arm		Right homologous arm		Target site of sgRNA	
			Forward primer	Reverse primer	Forward primer	Reverse primer	Forward oligo	Reverse oligo
Rbpm1	PY17X_0716700	N-terminal 4Myc	CGGGGTACCGCATACACGAGGAAATACTA	CATGCCATGGTTTGTGTATTTTATTTTTCGTCG	CCGCTCGAGTTTATGTCATTTTTTGAGGT	CCGGAATTCGTTCCAATGAAGACAAACAA	TATTGCGGACGCTAATCGTAGCTA	AAACTAGCTACGATTAGCGTCCGC

Diagnostic PCR primers for *in situ* complementation gene

Strain	P1	P2	P3	P4				
rescue	AAGCGAAATAAATGAAACGG	GGTTAAAAGCTAAAAAGGCC	CGACGAAAAATAAAATACACAAA	CGTAATTTCAAAAATGAAGA				

Oligo sequences for constructing intron deletion plasmids

Strain	Homologous arm		Mutation primers		Target site of sgRNA	
	Forward primer	Reverse primer	Forward primer	Reverse primer	Forward oligo	Reverse oligo
kinesin8bΔI1	CATGCCATGGTGATAAAGAAAAAACACACG	CCGCTCGAGCATGTTTAGGTAGATAATGT	ACTCAACTAAAGGAAAACATAACAGATAAAGACAAT	TGTTATGTTTTCCTTTAGTTGAGTGTATTTGCGTTT	TATTGTGACGCTTGACTAACGATG	AAACCATCGTTAGTCAAGCGTCAC
PF16ΔI1	CGGGGTACCCCAGCTATATTATGTCTTGG	CCGCTCGAGTTCTTCGGTATTTTCAACTTCATCA	TCAGGGTGCTTAAAATATTTGCAAGTAATTTTTATC	AAATACAATGGGGATAAAAATTACTTGCAAATATTT	TATTGTGTGTACATACTTCATGTG	AAACCACATGAAGTATGTACACAC
dlc1ΔI4	CGGGGTACCGGCCATACTATTTATAAACA	CCGCTCGAGATGCTCTATAAAAATACAGC	TCAAATACATTATAAGTTGCTACATTTTAAAGAATT	TGTAGCAACTTATAATGTATTTGAGAGGAAGCATTT	TATTGCATTCTTCACAAATTATGT	AAACACATAATTTGTGAAGAATGC

1109100 Δl1	CGGGGTACCGTACGTACATGTAGGACAAA	CCGGAATTCGTGGGAAAAAATAAGTATTAC	GTTGAGCGCGGGTTCTAAGCCCTTAGAAATATGGAA		AAGGGCTTAGAACCCGCGCTCAACAAGAGTGACAAT		TATTGGAAAATGAGAACAAAAAAA	AAACTTTTTTTGTTCTCATTTTCC	
**Diagnostic PCR primers for intron deletion**									
**Strain**	**P1**	**P2**	**P3**	**P4**	**P5**	**P6**			
kinesin8b Δl1	CTGAAATAGAATGCTTAAAAGAAC	GCATTGTCTTTATCTGTTATGTT	AATTTGGGTTCTATAACCCC	CGCTCATTTAAAACCTGTTTAC	GGGGCTAGTTAGATCGTACC	CGGATAATGAACCATCGATT			
PF16 Δl1	GTACGTGCAGGTGCTTTACC	CCTCACATGAAGTATGTACA	CATTTATTAAATCAGGGTGC	GCACAATAATGTATTGGAAA	CCTAGTTGTAATTTCTCAAA	ACGATTTGGTTATATATAAT			
dlc1 Δl4	GCACACGTAATCAAATTTAA	TCTTTAAAATGTAGCAACTTA	GCTTCCTCTCAAATACATTA	CCATCTGATACTTTCAATGC	TTCAAAAAGTAGAAGTATGC	CTGAAAAGTACGTTATAAGG			
1109100 Δl1	CATATGCAAATAATATTTGCAC	TATTTCTAAGGGCTTAGAAC	ATTGTCACTCTTGTTGAGCG	TATTCATCTAAGCAGAAAAAT	CGGTAGCAGTATATGTAGTT	CTGATTATAAAAGGAAAGAC			
**Oligo sequences for constructing *bfp* reproter assay plasmids**									
**Expression element**	**Forward primer**	**Reverse primer**	**Reporter gene**	**Forward primer**	**Reverse primer**	**Expression element**	**Forward primer**	**Reverse primer**	
The 5'-UTR of *hsp70*	CGGGGTACCGTAAAGAGGATGATGTATGT	CATGCCATGGCTTTTTTTTTTTTTAATTGCAA	*bfp*	CATGCCATGGGGATGGTGAGCAAGGGCGAGGAGCT	CCGCTCGAGTTACTTGTACAGCTCGTCCATGC	The 3'-UTR of *dhfr*	CCGCTCGAGTGTTCATTTTTCTTATTTAT	CCGGAATTCCCTGAAGAAGAACAGTCCGA	
**Reporter gene**	**Upstream *bfp* sequence**		**Intron**		**Downstream *bfp* sequence**		**Overlap PCR**		
	**Forward primer**	**Reverse primer**	**Forward primer**	**Reverse primer**	**Forward primer**	**Reverse primer**	**Forward primer**	**Reverse primer**	
bfp-Kin8b I1	CATGCCATGGGGATGGTGAGCAAGGGCGAGGA	TAGCCCCAACCTTGAAGTCGACGCCCTTCA	CGACTTCAAGGTTGGGGCTAGTTAGATCGT	TGCCGTCCTCCTACAAATAAACAAAATAAC	TTATTTGTAGGAGGACGGCAACATCCTGGG	CCGCTCGAGTTACTTGTACAGCTCGTCCATGC	CATGCCATGGGGATGGTGAGCAAGGGCGAGGA	CCGCTCGAGTTACTTGTACAGCTCGTCCATGC	
bfp-Kin8b I2	CATGCCATGGGGATGGTGAGCAAGGGCGAGGA	CTGAATATACTTGTGGCCGTTTACGTCGCC	ACGGCCACAAGTATATTCAGTTCTTTCCAT	CACGCTGAACCTGTTTACATATATTAAAAA	ATGTAAACAGGTTCAGCGTGAGGGGCGAGG	CCGCTCGAGTTACTTGTACAGCTCGTCCATGC	CATGCCATGGGGATGGTGAGCAAGGGCGAGGA	CCGCTCGAGTTACTTGTACAGCTCGTCCATGC	
bfp-PF16 I1	CATGCCATGGGGATGGTGAGCAAGGGCGAGGA	TTTATTTTACTTGAAGTTCACCTTGATGCC	TGAACTTCAAGTAAAATAAAACAAAACAAA	GTGGCGGATCCTAAATAAAAGAGAAAAGCC	TTTTATTTAGGATCCGCCACAACGTGGAGG	CCGCTCGAGTTACTTGTACAGCTCGTCCATGC	CATGCCATGGGGATGGTGAGCAAGGGCGAGGA	CCGCTCGAGTTACTTGTACAGCTCGTCCATGC	
bfp-dlc1 I4	CATGCCATGGGGATGGTGAGCAAGGGCGAGGA	AATATGTTACTAGATGTTGTGGCTGTTGAA	ACAACATCTAGTAACATATTAATATAATTT	GGCCATGATACTGAAAAGTACGTTATAAGG	TACTTTTCAGTATCATGGCCGTCAAGCAGA	CCGCTCGAGTTACTTGTACAGCTCGTCCATGC	CATGCCATGGGGATGGTGAGCAAGGGCGAGGA	CCGCTCGAGTTACTTGTACAGCTCGTCCATGC	
bfp-1109100 I1	CATGCCATGGGGATGGTGAGCAAGGGCGAGGA	ATTAAATTACGTCGACGCCCTTCAGCTCGA	GGGCGTCGACGTAATTTAATATTATAATAA	CCTCCTTGAACTGATTATAAAAGGAAAGAC	TTATAATCAGTTCAAGGAGGACGGCAACAT	CCGCTCGAGTTACTTGTACAGCTCGTCCATGC	CATGCCATGGGGATGGTGAGCAAGGGCGAGGA	CCGCTCGAGTTACTTGTACAGCTCGTCCATGC	
bfp-1109100 I2	CATGCCATGGGGATGGTGAGCAAGGGCGAGGA	TAAGTATTACGTCGACGCCCTTCAGCTCGA	GGGCGTCGACGTAATACTTATTTTTTCCCA	CCTCCTTGAACTAAGCAGAAAAATTTTAAA	TTCTGCTTAGTTCAAGGAGGACGGCAACAT	CCGCTCGAGTTACTTGTACAGCTCGTCCATGC	CATGCCATGGGGATGGTGAGCAAGGGCGAGGA	CCGCTCGAGTTACTTGTACAGCTCGTCCATGC	
**Diagnostic PCR primers for expression cassette integrated into the *p230p* locus**									
**P1**	**P2**	**P3**	**P4**	**P5**	**P6**				
GGAAAAGTATGATAACGATG	GACGGACACATACATCATCC	TCGGACTGTTCTTCTTCAGG	AGATGATATCGCTATATATC	GATGCATCTATAACTCCAGA	TGCTGAGTCAGTGGTGTTCC				
**Oligo sequences for constructing plasmids with axonemal intron insertion into *gep1***									
**Strain**	**Upstream *gep1* sequence**		**Intron**		**Downstream *gep1* sequence**		**Overlap PCR**		
	**Forward primer**	**Reverse primer**	**Forward primer**	**Reverse primer**	**Forward primer**	**Reverse primer**	**Forward primer**	**Reverse primer**	
gep1-Kin8b I1	CGGGGTACCGTCAAATCATGAAGTTTGGA	TAGCCCCAACCTTTTTGATGCTGATATAAG	CATCAAAAAGGTTGGGGCTAGTTAGATCGT	TACATCTATCCTACAAATAAACAAAATAAC	TTATTTGTAGGATAGATGTATTATTTTTGT	CATGCCATGGCTTTGAGGAGAGAGTGTGGG	CGGGGTACCGTCAAATCATGAAGTTTGGA	CATGCCATGGCTTTGAGGAGAGAGTGTGGG	
gep1-PF16 I1	CGGGGTACCATGTGTTGAAATAAAAGATG	TTTATTTTACTTGCAATAAAATGGAGCCG	TTTATTGCAAGTAAAATAAAACAAAACAAA	CATTAATCTCCTAAATAAAAGAGAAAAGCC	TTTTATTTAGGAGATTAATGGAGGAAGATT	GGGCTTAAGGTAGACTTACCAACTTCATT	CGGGGTACCATGTGTTGAAATAAAAGATG	GGGCTTAAGGTAGACTTACCAACTTCATT	
**Strain**	**Target site of sgRNA**								
	**Forward oligo**	**Reverse oligo**							
gep1-Kin8b I1	TATTGTATTGATTCCGAGTCTGAAA	AAACTTTCAGACTCGGAATCAATAC							
gep1-PF16 I1	TATTGTTATTGCAAGAGATTAATGG	AAACCCATTAATCTCTTGCAATAAC							
**Diagnostic PCR primers for axonemal intron inserted into *gep1***									
**Strain**	**P1**	**P2**	**P3**	**P4**					
gep1-Kin8 I1	TATTGAGCTACTGTCAGCAG	ACAAAAATAATACATCTATC	GCTTATATCAGCATCAAAAAG	CTCATTTTTCGGCTTATCAC					
gep1-PF16 I1	GTGATGTACGAAAAATTCGA	GGCAAGTGTTTATAGAATTG	CGGCTCCATTTTTATTGCAA	GAAAGCAATGATGTCTCATCC					
**Primers for RT-PCR**									
**Primer name**	**Primer sequence**	**Primer name**	**Primer sequence**		**Primer name**	**Primer sequence**	**Primer name**	**Primer sequence**	
kinesin8b F1	CAAAGTTAATCTTCAAGAGT	*kinesin8b* R1	AGTTCATCTTGCAATCCTTC		PY17X_1357300 F6	GGAATTTGCAGAAAGTACCC	PY17X_1357300 R6	CGATTTTTTTTCCGATTTTT	
kinesin8b F2	GAAAACATAACAGATAAAGA	*kinesin8b* R2	CTCCTCAAGCATCTTAATAT		PY17X_1357300 F7	GGAGAAGCCACTCAACTTAT	PY17X_1357300 R7	GTATCATTAAACATGGAATC	
PF16 F1	CATTTATTAAATCAGGGTGC	*PF16* R1	TCGGTATTTTCAACTTCATC		PY17X_1335600 F1	CAATGAAGAAGGAAATTCCG	PY17X_1335600 R1	GAACTATCCGAGCAACTCTC	
dhc6 F19	CAAGAAAATGATGCATTAAA	*dhc6* R19	GTAGCGTTGATTTTCCACTA		PY17X_1452900 F1	CAGCCTGAATATAAACCTAA	PY17X_1452900 R1	ATATGCTGTTTAAACTCCAC	
dhc6 F20	TTTCTACAACAAGAGAGAAT	*dhc6* R20	GGAGTCATATAATTGATCTT		PY17X_1452900 F2	GTGGAGTTTAAACAGCATAT	PY17X_1452900 R2	ATCAGCAAAACTTATGTGGG	
dhc7 F6	GAGGCAATTATGATGACGTG	*dhc7* R6	CTTCAAAACATGATTTAACA		PY17X_1122300 F1	GCCTTTTTAGCTATTTTTGTG	PY17X_1122300 R1	GTAGAATCCGAAGTAATTCG	
dhc7 F7	CCTACTAATCTTATAACATT	*dhc7* R7	TTTGTATCTCAATAAGCTTC		PY17X_1122300 F2	CGAATTACTTCGGATTCTAC	PY17X_1122300 R2	TTCCATTTATGATAATCGTC	
dhc7 F8	ATCTGATCCAAGAATACAAC	*dhc7* R8	GCTCACGAATATTAGTTCCC		PY17X_0523500 F1	TAACACCATATAACAATAAC	PY17X_0523500 R1	ATAATGTGAAAAGATCTTCC	
dlc1 F3	ACGTTTTAAAGAACAAAATG	*dlc1* R3	TAATGTATTTGAGAGGAAGC		PY17X_0523500 F2	CACCACATCTATCTTACCAG	PY17X_0523500 R2	GGGATCCAGAATTGCAAGGT	
dlc1 F4	TGGTCCAACATAATTTGTGAAG	*dlc1* R4	TGAATAGAAGTCTCTGCATTCG		PY17X_0523500 F3	GACAATATTCCAGACACTAA	PY17X_0523500 R3	AATTTGTGGAAATGTCCAGA	
dlc1 F5	GCTACATTTTAAAGAATTCG	*dlc1* R5	GCACAATATATATGAGCATG		PY17X_0508900 F4	CAAATTCCGATTTTTACGAT	PY17X_0508900 R4	CCTTCTTTTTACTCATTAAT	
dlc2 F1	ATGAGTTCCGAAAATTTTTC	*dlc2* R1	TTCAACTTGTATAGCATTTG		PY17X_0508900 F5	AGACAGAGGACTTAATTCAG	PY17X_0508900 R5	GTGTGTCATCAATTAGTTGT	
dlc2 F2	AAATCTAATATTCCTTATCA	*dlc2* R2	CTTTGCTGAGATGTTAAACG		PY17X_0508900 F6	GCTTTCTTATGATGTTAACA	PY17X_0508900 R6	CGCATTTGCTTTCTTCGCTC	
drc1 F1	ATGCTACAAAACAATCTTAA	*drc1* R1	TTTCAAATTTCTTAACAGAC		PY17X_1320300 F1	GGGAAGATAACATTGAGGAG	PY17X_1320300 R1	TATGTACTAGTTTTTTCTGC	
drc1 F2	GTCCAACCACTTTAAGATGG	*drc1* R2	CTATTAACACATTTACACTGC		PY17X_1320300 F2	CTCATGAAAATTTGGAAGAG	PY17X_1320300 R2	CTCGTCATGTGATTGCTTAA	
drc1 F3	ATTTTGAAGATGATCAAGAG	*drc1* R3	CGTTTTATCATTTTCTTCCT		PY17X_1320300 F3	AAAATATTAAAATTGAAAGC	PY17X_1320300 R3	CATTCATGAGTTTCTTTTGA	
dbc F1	CGCAGCGGATAATATCCAAC	*dbc* R1	TTTCAAAATTTACTTCCTCC		PY17X_0833600 F1	CAGATGGAGTATTAATGGCA	PY17X_0833600 R1	TTTAAAGGGTCGGGGTTCTG	
dbc F2	GAGTTATCAAAAACACTGGAT	*dbc* R2	CGTACTTTAATTTACGTATCC		PY17X_0833600 F2	CAGAACCCCGACCCTTTAAA	PY17X_0833600 R2	TATTTAATGCCTCAATTTTC	
md2 F1	CTAGCTAGAGCTATGCGTAA	*md2* R1	TCAATAGGAACTTGTGAATC		PY17X_1341200 F1	GCAGTAATGACAATAGAAGT	PY17X_1341200 R1	GGATGTCGAGTTTTTATCCT	
PY17X_1109100 F1	TTGTCACTCTTGTTGAGCGC	PY17X_1109100 R1	GCTGTGTATATGTACGAGTC		PY17X_1341200 F2	AGGATAAAAACTCGACATCC	PY17X_1341200 R2	TACAAATGATACTTTTTGCG	
PY17X_1109100 F2	GGGTGTGTTAGAAAAGTTTT	PY17X_1109100 R2	TTCGAAAAGTTCTTCGACAC		PY17X_1305400 F10	GAGATTTGTACGAATGTCGA	PY17X_1305400 R10	CCTGACTCCATATTATATCT	
PY17X_0521800 F1	CGAAACTGATGTTAAAAATA	PY17X_0521800 R1	CTCGTCAGAATTAAAGTATT		PY17X_1305400 F12	CACAAAGAAGGCAATCATGT	PY17X_1305400 R12	GTCATTGATAAGTTGATCTG	
PY17X_0521800 F2	CGCAGTAAATATAGAGTTAG	PY17X_0521800 R2	TATAAAAACTTCTTCGTCAG		PY17X_1305400 F13	GATTCTGAGGAATTTGATCA	PY17X_1305400 R13	CATCATTTAGTTCTTTGCAA	
PY17X_1311800 F4	ATAGTGTTTTATCCTAAATC	PY17X_1311800 R4	GTCTTCTATTTCTCCACTCT		PY17X_0415900 F12	CGTTGAAAATGTTTCCTCAA	PY17X_0415900 R12	TCTTATGCATTTTTATCTCC	
PY17X_1311800 F5	GATATTACCCATAAGGTGTG	PY17X_1311800 R5	CGTAGCTAAATATCATGTAG		PY17X_0415900 F13	GCCACATTTTTACGAAAATG	PY17X_0415900 R13	AGTATGTCTCTGGCCCTTAT	
PY17X_1311800 F6	ATTAAGATTGTATTCCTTCC	PY17X_1311800 R6	ATATCTATATTCATAGACCC		PY17X_0105800 F1	GCTACCTGTCTTATTTTATC	PY17X_0105800 R1	ACGTCCTCAAACTTGAGCTC	
PY17X_1323900 F1	GTATTTGCTCGACAAAAATG	PY17X_1323900 R1	GTCTTTCCAGTTTTATACAC		PY17X_1216400 F4	GAGAGGAAACATAAATTTGTC	PY17X_1216400 R4	CTTTTCTAACTCATAAGTTTG	
PY17X_1357300 F5	AATATAAGCATCTATCACAG	PY17X_1357300 R5	GGATCGTTTTATGTATCCAA		PY17X_1216400 F5	CGCAGACATTAAAAAGAGGG	PY17X_1216400 R5	ATCGTTTCCTTCTGTTTCAG	
**Primers for RT-qPCR**									
**Location**	**Gene ID**	**Forward primer**	**Reverse primer**		**Location**	**Gene ID**	**Forward primer**	**Reverse primer**	
GAPDH	PY17X_1330200	GAGCAGGTAGATCAGCTTTGT	ACTCTAAAGGCAACACCAGTTA		*1216400*	PY17X_1216400	CAACCCTGAAGAGATACCTCAAA	CCCGTTATCTTCTTCATCAGCA	
α-tubulin 2	PY17X_0524100	ATGTTGGTCAAGCAGGTATCC	CAACTTGATCACTGGGCATTTG		*kinesin8b* I1	PY17X_0204100	GGCTAGTTAGATCGTACCACTTC	TGGCGCTTGACTAACGATAT	
β-tubulin	PY17X_1210100	CGGAGAAGGTATGGACGAAATG	CTTCAGCAGTGGCATCTTGATA		*kinesin8b* I4	PY17X_0204100	AAGCAAGGAAAATGCAACGC	GAGAGTTAAACAATGAGCAG	
Rbpm1	PY17X_0716700	CGCACAAGATCACGAGACAA	CATCCACTCGTTTCCCATTCA		*PF16* I1	PY17X_0919000	ATTCCTAGTTGTAATTTCTC	CCAATGTGTAAATATTGCAT	
1109100	PY17X_1109100	TTGTCACTCTTGTTGAGCGC	GCTGTGTATATGTACGAGTC		*PF16* I1/E2	PY17X_0919000	GTGTGTACATACTTCATGTG	CGAATTTCGTGGAATCTTCAC	
0833600	PY17X_0833600	CATGGGTGTGGAAAAAGTCC	GGGATTTCCGTTTCATGACT						
**Primers for RIP-qPCR**									
**Location**	**Gene ID**	**Forward primer**	**Reverse primer**		**Location**	**Gene ID**	**Forward primer**	**Reverse primer**	
kinesin8b I1	PY17X_0204100	GGCTAGTTAGATCGTACCACTTC	TGGCGCTTGACTAACGATAT		*dlc1* I4	PY17X_1241500	TTCAAAAAGTAGAAGTATGC	GTGATAGTTGTGCAAATAATAC	
kinesin8b I4	PY17X_0204100	AAGCAAGGAAAATGCAACGC	GAGAGTTAAACAATGAGCAG		*dlc1* I3	PY17X_1241500	GTGCATATCCTACACGGTTG	GGTGATACACACATTACTAT	
PF16 E1	PY17X_0919000	GCTAGCTAATATTGTTGCTGAAGA	GATTGCACACAATGCGTTAATAC		*dlc2* I1	PY17X_0302800	GAAACAATAATAAGCGAAATCG	TCACATACATACTTTTTTAAC	
PF16 I1	PY17X_0919000	GAATAATTTAACCATCTTTTTCAC	CCCTACGATTTGGTTATATATA		*dlc2* I3	PY17X_0302800	GTTAGAGCATGCTTTATAGA	TGAAGTAAAATTGTGACACC	
dhc6 I20	PY17X_0603800	CAATCCTAATAGGCACCCCC	TATATTTGTCTGTATGAACG		PY17X_1109100 I1	PY17X_1109100	CGGTAGCAGTATATGTAGTT	CTAGTTCAATGTTTTCTACA	
dhc6 E22	PY17X_0603800	CCTCCTATAGACGAATCTAA	CTCTTTCTGCTTGTGATATAC		PY17X_1109100 I2	PY17X_1109100	CCCACTTTTTTCAATTCCCC	CAGGTATATTTATGCGTATAC	
**Primers for obtaining *in vitro* transcription templates**									

Probe	Gene ID	Forward primer	Reverse primer		Probe	Gene ID	Forward primer	Reverse primer
kinesin8b I4 probe	PY17X_0204100	TAATACGACTCACTATAGGGAGACTAAAATGGAAAACCAGTTAC	TAAGGTGAATGGTAAAGTTC		*bfp*	/	TAATACGACTCACTATAGGGAGAATGGTGAGCAAGGGCGAGGA	CTTGTACAGCTCGTCCATGC
kinesin8b I1 probe	PY17X_0204100	TAATACGACTCACTATAGGGAGAATGAAAAATTATTTATAGAC	TAAGTTCATCTTGCAATCCT		*bfp-Kin8b* I1	/	TAATACGACTCACTATAGGGAGAATGGTGAGCAAGGGCGAGGA	CTTGTACAGCTCGTCCATGC
PF16 E1 probe	PY17X_0919000	TAATACGACTCACTATAGGGAGAAGATAAAGTTCCTATAGTTC	AAAATTTGATTAAATTTGGT		*bfp-PF16* I1	/	TAATACGACTCACTATAGGGAGAATGGTGAGCAAGGGCGAGGA	CTTGTACAGCTCGTCCATGC
PF16 I1 probe	PY17X_0919000	TAATACGACTCACTATAGGGAGACTAAAATCACCTTTAAAACT	TCAGAATATCCAGGTGTGTA					

Note: The blue sequences are designed for the restriction enzyme digestion. The red sequences are T7 promoter sequence.
