# Supplementary material for: An axonemal intron splicing program sustains Plasmodium male development
Source: Nat Commun. 2024 Jun 1;15:4697. doi: 10.1038/s41467-024-49002-9 (PMC11144265; doi:10.1038/s41467-024-49002-9)
Supplement: Supplementary file 3 — Description of Additional Supplementary Files [file 41467_2024_49002_MOESM3_ESM.docx]

**Description of additional supplementary data files**

**An axonemal intron splicing program sustains *Plasmodium*** **male development**

Jiepeng Guan^1,4^, Peijia Wu^1,4^, Xiaoli Mo^1,4^, Xiaolong Zhang^3,4^, Wenqi Liang^1^, Xiaoming Zhang^1^, Lubin Jiang^3,^*, Jian Li^1,^*, Huiting Cui^1,^* and Jing Yuan^1,2,^*

**1.**

**Title:** Supplementary Data 1

**Description:** List of differentially expressed genes between male and female gametocytes of the *P.yoelii DFsc7* line

**2.**

**Title:** Supplementary Data 2

**Description:** List of differentially expressed genes in male gametocytes between *DFsc7* (Parental) and *DFsc7*;Δ*Rbpm1* (Mutant) lines of the *P.yoelii*

**3.**

**Title:** Supplementary Data 3

**Description:** List of the RBPm1 interacting protein candidates identified in this study
