# Supplementary material for: An axonemal intron splicing program sustains Plasmodium male development
Source: Nat Commun. 2024 Jun 1;15:4697. doi: 10.1038/s41467-024-49002-9 (PMC11144265; doi:10.1038/s41467-024-49002-9)
Supplement: Supplementary file 7 — Reporting Summary [file 41467_2024_49002_MOESM7_ESM.pdf]

nature portfolio

Corresponding author(s): Jing Yuan

Last updated by author(s): Apr 22, 2024

Reporting Summary

Nature Portfolio wishes to improve the reproducibility of the work that we publish. This form provides structure for consistency and transparency in reporting. For further information on Nature Portfolio policies, see our Editorial Policies and the Editorial Policy Checklist.

Statistics

For all statistical analyses, confirm that the following items are present in the figure legend, table legend, main text, or Methods section.

n/a	Confirmed	
☐	☒	The exact sample size (n) for each experimental group/condition, given as a discrete number and unit of measurement
☐	☒	A statement on whether measurements were taken from distinct samples or whether the same sample was measured repeatedly
☐	☒	The statistical test(s) used AND whether they are one- or two-sided<br>*Only common tests should be described solely by name; describe more complex techniques in the Methods section.*
☒	☐	A description of all covariates tested
☒	☐	A description of any assumptions or corrections, such as tests of normality and adjustment for multiple comparisons
☐	☒	A full description of the statistical parameters including central tendency (e.g. means) or other basic estimates (e.g. regression coefficient) AND variation (e.g. standard deviation) or associated estimates of uncertainty (e.g. confidence intervals)
☐	☒	For null hypothesis testing, the test statistic (e.g. F, t, r) with confidence intervals, effect sizes, degrees of freedom and P value noted<br>*Give P values as exact values whenever suitable.*
☒	☐	For Bayesian analysis, information on the choice of priors and Markov chain Monte Carlo settings
☒	☐	For hierarchical and complex designs, identification of the appropriate level for tests and full reporting of outcomes
☐	☒	Estimates of effect sizes (e.g. Cohen's d, Pearson's r), indicating how they were calculated

Our web collection on statistics for biologists contains articles on many of the points above.

Software and code

Policy information about availability of computer code

Data collection	Flow cytometry data were collected using BD LSRFortessa and BD FACS AriaIII flow cytometers.<br>Fluorescent images were acquired using Zeiss LSM 780 and Zeiss LSM 980 confocal microscopes.<br>Electron microscopy was conducted using the Hitachi HT-7800 electron microscope.<br>DNA gels were imaged with the Gel Image System (Tanon-2500).
Data analysis	Flow cytometry data were analyzed using FlowJo (version 10.7.1).<br>Fluorescent images were processed with ZEN 3.4 (blue edition).<br>Statistical analysis was conducted using GraphPad Prism (version 8.0.2).<br>The codes, scripts and supporting files for the bioinformatic analysis of global intron retention were uploaded to GitHub at https://github.com/xiaolimo29/Intron_retention.<br>The protein signal on the blotting membrane was quantified using ImageJ (version 1.54f).<br><br>The following analysis packages were used for bioinformatic analysis (specific functions are specified in the methods):<br>Trim Galore (v0.6.10)<br>HISAT2 (v2.2.1)<br>SAMtools (v1.16.1)<br>featureCounts (v2.0.3)<br>t-arae/ngscmdr (v0.1.0.181203)<br>edgeR (v3.40.2)

BEDOPS (v2.4.41)
DeepTools (v3.5.1)
Integrative Genomics Viewer (v2.16.1)
Cufflinks (v2.2.1)

For manuscripts utilizing custom algorithms or software that are central to the research but not yet described in published literature, software must be made available to editors and reviewers. We strongly encourage code deposition in a community repository (e.g. GitHub). See the Nature Portfolio guidelines for submitting code & software for further information.

Data

Policy information about availability of data

All manuscripts must include a data availability statement. This statement should provide the following information, where applicable:

- Accession codes, unique identifiers, or web links for publicly available datasets
- A description of any restrictions on data availability
- For clinical datasets or third party data, please ensure that the statement adheres to our policy

RNA-seq data for the P. yoelii male- and female-specific gametocyte transcriptome is available via the Gene Expression Omnibus database under the accession number GSE222860.
RNA-seq data for male gametocyte transcriptome of the P. yoelii Rbpm1 knockout parasite line is available under accession number GSE223170.
The mass spectrometry proteomic data can be accessed through ProteomeXchange with identifier PXD044094.

Research involving human participants, their data, or biological material

Policy information about studies with human participants or human data. See also policy information about sex, gender (identity/presentation), and sexual orientation and race, ethnicity and racism.

Reporting on sex and gender	N/A no Human subjects
Reporting on race, ethnicity, or other socially relevant groupings	N/A no Human subjects
Population characteristics	N/A no Human subjects
Recruitment	N/A no Human subjects
Ethics oversight	N/A no Human subjects

Note that full information on the approval of the study protocol must also be provided in the manuscript.

Field-specific reporting

Please select the one below that is the best fit for your research. If you are not sure, read the appropriate sections before making your selection.

[x] Life sciences [ ] Behavioural & social sciences [ ] Ecological, evolutionary & environmental sciences

For a reference copy of the document with all sections, see nature.com/documents/nr-reporting-summary-flat.pdf

Life sciences study design

All studies must disclose on these points even when the disclosure is negative.

Sample size	The sample size was determined based on similar experiments previously published in the lab, including "EB1 decoration of microtubule lattice facilitates spindle-kinetochore lateral attachment in Plasmodium male gametogenesis" (Nature Communications, 2023); "Apical anchorage and stabilization of subpellicular microtubules by apical polar ring ensures Plasmodium ookinete infection in mosquito" (Nature Communications, 2022); "A malaria parasite phospholipid flippase safeguards midgut traversal of ookinetes for mosquito transmission" (Science Advances, 2021). The sample sizes were considered sufficient to conduct the experiments with adequate statistical power using t-tests and other tests.
Data exclusions	In the bioinformatic analysis of global intron retention, low-expressed genes (TPM below 30) were excluded. To eliminate potential false positives, introns with peak scores exceeding 50% of adjacent exons were discarded in parental parasites. In mutant parasites, introns with peak scores below 50% of neighboring exons were also omitted.
Replication	All measurements were replicated biologically. The number of biological replicates is stated in each figure legend. All attempts at replication were successful. The mosquito infection experiment was performed once for some modified (gene tagging) parasites in Supplementary Table 1, which is sufficient to confirm the normal life cycle progression for these parasites.
Randomization	For the parasite infection assay, mice and mosquitoes were randomly divided into corresponding groups.
Blinding	In this study, there were not any experiment which required the blinding of the samples.

Reporting for specific materials, systems and methods

We require information from authors about some types of materials, experimental systems and methods used in many studies. Here, indicate whether each material, system or method listed is relevant to your study. If you are not sure if a list item applies to your research, read the appropriate section before selecting a response.

Materials & experimental systems

n/a	Involved in the study
☐	☒ Antibodies
☒	☐ Eukaryotic cell lines
☒	☐ Palaeontology and archaeology
☐	☒ Animals and other organisms
☒	☐ Clinical data
☒	☐ Dual use research of concern
☒	☐ Plants

Methods

n/a	Involved in the study
☒	☐ ChIP-seq
☐	☒ Flow cytometry
☒	☐ MRI-based neuroimaging

Antibodies

Antibodies used	The following primary antibodies were utilized: rabbit anti-HA (Cell Signaling Technology, cat#3724S; IFA, 1:1000 dilution; IB, 1:1000 dilution), rabbit anti-mCherry (Abcam, cat# ab167453; IFA, 1:1000 dilution), rabbit anti-histone H3 antibody (Abcam, cat#ab1791; IFA, 1:1000 dilution), rabbit anti-Myc (Cell Signaling Technology, cat#2276S; IFA, 1:1000 dilution; IB, 1:1000 dilution), mouse anti-α-Tubulin (Sigma-Aldrich, cat#T6199; IFA, 1:1000 dilution; IB, 1:1000 dilution; U-ExM, 1:500 dilution), mouse anti-β-Tubulin (Sigma-Aldrich, cat#T5201; IB, 1:1000 dilution) and mouse anti-HA (Santa Cruz Biotechnology, cat#sc-57592; IFA, 1:200 dilution). The secondary antibodies included: Alexa Fluor 555 goat anti-rabbit IgG (Thermo Fisher Scientific, cat#A-21428; IFA, 1:1000 dilution), Alexa Fluor 488 goat anti-rabbit IgG (Thermo Fisher Scientific, cat#A-31566; IFA, 1:1000 dilution), Alexa Fluor 555 goat anti-mouse IgG (Thermo Fisher Scientific, cat# A-21422; IFA, 1:1000 dilution; U-ExM, 1:500 dilution), Alexa Fluor 488 goat anti-mouse IgG (Thermo Fisher Scientific, cat#A-11001; IFA, 1:1000 dilution), Alexa Fluor 488 goat anti-mouse TER-119 (BioLegend, cat#116215; IFA, 1:500 dilution), Alexa Fluor 488 conjugated streptavidin (Invitrogen, cat# S32354 ; IFA, 1:1000 dilution), HRP-conjugated goat anti-rabbit IgG (Abcam, cat#ab6721; IB, 1:5000 dilution) and HRP-conjugated goat anti-mouse IgG (Abcam, cat#ab6789; IB, 1:5000 dilution). The antiserum, including rabbit anti-BiP (IB, 1:1000 dilution) and rabbit anti-P28 (IFA, 1:1000), were previously in-house prepared in the laboratory.
Validation	All antibodies were obtained commercially. These antibodies were tested and validated by the respective company. All antibodies had validation statement provided on the website of the manufacturer.<br>rabbit anti-HA (Cell Signaling Technology, cat#3724S) https://www.cellsignal.com/products/primary-antibodies/ha-tag-c29f4-rabbit-mab/3724<br>rabbit anti-mCherry (Abcam, cat# ab167453) https://www.abcam.com/products/primary-antibodies/mcherry-antibody-ab167453.html<br>rabbit anti-histone H3 antibody (Abcam, cat#ab1791) https://www.abcam.cn/products/primary-antibodies/histone-h3-antibody-nuclear-marker-and-chip-grade-ab1791.html?productWallTab=ShowAll<br>rabbit anti-Myc (Cell Signaling Technology, cat#2276S) https://www.cellsignal.cn/products/primary-antibodies/myc-tag-antibody/2272?<br>mouse anti-α-Tubulin (Sigma-Aldrich, cat#T6199) https://www.sigmaaldrich.cn/CN/zh/product/sigma/t6199<br>mouse anti-β-Tubulin (Sigma-Aldrich, cat#T5201) https://www.sigmaaldrich.cn/CN/zh/product/sigma/t5201<br>mouse anti-HA (Santa Cruz Biotechnology, cat#sc-57592) https://www.scbt.com/p/ha-probe-antibody-12ca5<br>rabbit anti-BiP and rabbit anti-P28 doi: 10.1016/j.cub.2018.06.069

Animals and other research organisms

Policy information about studies involving animals; ARRIVE guidelines recommended for reporting animal research, and Sex and Gender in Research

Laboratory animals	Female ICR mice aged 5-6 weeks were acquired from the Animal Care Center of Xiamen University. The mice were housed in a controlled environment at 22-24°C, relative humidity of 45–65%, and a 12-hour light/dark cycle.<br>The larvae of Anopheles stephensi mosquitoes (Hor strain) were maintained in an insect facility under controlled conditions of 28°C, 80% relative humidity, and a 12-hour light/12-hour dark cycle. Adult mosquitoes were fed with a 10% (w/v) sucrose solution containing 0.05% 4-aminobenzoic acid and kept at 23°C.
Wild animals	The study did not use wild animals.
Reporting on sex	Female mice were used for the Plasmodium yoelii parasite infection. Up to five mice per cage were arranged, which make it easier and more cost-effective to maintain.<br>For mosquito infection via mouse blood feeding, only female mosquitoes were used.
Field-collected samples	No field-collected samples were used in this study.

Ethics oversight	All experiments were reviewed and approved by the Committee for Care and Use of Laboratory Animals of Xiamen University (XMULAC20190001).

Note that full information on the approval of the study protocol must also be provided in the manuscript.

Plants

Seed stocks	*Report on the source of all seed stocks or other plant material used. If applicable, state the seed stock centre and catalogue number. If plant specimens were collected from the field, describe the collection location, date and sampling procedures.*
Novel plant genotypes	*Describe the methods by which all novel plant genotypes were produced. This includes those generated by transgenic approaches, gene editing, chemical/radiation-based mutagenesis and hybridization. For transgenic lines, describe the transformation method, the number of independent lines analyzed and the generation upon which experiments were performed. For gene-edited lines, describe the editor used, the endogenous sequence targeted for editing, the targeting guide RNA sequence (if applicable) and how the editor was applied.*
Authentication	*Describe any authentication procedures for each seed stock used or novel genotype generated. Describe any experiments used to assess the effect of a mutation and, where applicable, how potential secondary effects (e.g. second site T-DNA insertions, mosiacism, off-target gene editing) were examined.*

Flow Cytometry

Plots

Confirm that:

- [x] The axis labels state the marker and fluorochrome used (e.g. CD4-FITC).
- [x] The axis scales are clearly visible. Include numbers along axes only for bottom left plot of group (a 'group' is an analysis of identical markers).
- [x] All plots are contour plots with outliers or pseudocolor plots.
- [x] A numerical value for number of cells or percentage (with statistics) is provided.

Methodology

Sample preparation	The sample preparation details are provided in the methods section. Briefly, to analyze DNA content of male gametocytes, parasites containing gametocytes from mouse tail blood were stained with 4 µM Hoechst 33342 for 10 minutes at room temperature, followed by PBS washes. For sorting gametocytes, the purified gametocytes were directly sorted based on GFP and mCherry fluorescence for male and female gametocytes
Instrument	Flow cytometry analysis: BD LSRFortessa<br>Flow cytometry sorting: BD FACS AriaIII
Software	FACSDiva software (v9.0.1)
Cell population abundance	To analyze the DNA content of male gametocytes, male gametocytes are 2-6% of the population. For sorting gametocytes, the purified male gametocytes are 3-20% of the population before sorting, and after sorting, they are more than 95% of the population.
Gating strategy	To analyze the DNA content of male gametocytes, parasites containing gametocytes from the DFsc7 or DFsc7;ΔRbpm1 lines were staining with Hoechst 33342. Forward and side scatter signals, based on cell size and granularity, were used to distinguish red blood cells from debris, doublets and white blood cells. Male gametocytes were identified by GFP fluorescence and analyzed for Hoechst 33342 fluorescence. For sorting gametocytes, parasites containing gametocytes were sorted based on GFP and mCherry fluorescence for male and female gametocytes, respectively.

- [x] Tick this box to confirm that a figure exemplifying the gating strategy is provided in the Supplementary Information.
